# A Diverse Assemblage of Reef Corals Thriving in a Dynamic Intertidal Reef Setting (Bonaparte Archipelago, Kimberley, Australia)

**DOI:** 10.1371/journal.pone.0117791

**Published:** 2015-02-25

**Authors:** Zoe T. Richards, Rodrigo A. Garcia, Carden C. Wallace, Natalie L. Rosser, Paul R. Muir

**Affiliations:** 1 Department of Aquatic Zoology, Western Australian Museum, Welshpool, WA, 6016, Australia; 2 Remote Sensing and Satellite Research Group, Department of Imaging and Applied Physics, Curtin University, Bentley, WA, 6102, Australia; 3 Museum of Tropical Queensland, Flinders Street, Townsville, Qld, 4814, Australia; 4 School of Animal Biology, University of Western Australia, Crawley, WA, 6009, Australia; The Australian National University, AUSTRALIA

## Abstract

The susceptibility of reef-building corals to climatic anomalies is well documented and a cause of great concern for the future of coral reefs. Reef corals are normally considered to tolerate only a narrow range of climatic conditions with only a small number of species considered heat-tolerant. Occasionally however, corals can be seen thriving in unusually harsh reef settings and these are cause for some optimism about the future of coral reefs. Here we document for the first time a diverse assemblage of 225 species of hard corals occurring in the intertidal zone of the Bonaparte Archipelago, north western Australia. We compare the environmental conditions at our study site (tidal regime, SST and level of turbidity) with those experienced at four other more typical tropical reef locations with similar levels of diversity. Physical extremes in the Bonaparte Archipelago include tidal oscillations of up to 8 m, long subaerial exposure times (>3.5 hrs), prolonged exposure to high SST and fluctuating turbidity levels. We conclude the timing of low tide in the coolest parts of the day ameliorates the severity of subaerial exposure, and the combination of strong currents and a naturally high sediment regime helps to offset light and heat stress. The low level of anthropogenic impact and proximity to the Indo-west Pacific centre of diversity are likely to further promote resistance and resilience in this community. This assemblage provides an indication of what corals may have existed in other nearshore locations in the past prior to widespread coastal development, eutrophication, coral predator and disease outbreaks and coral bleaching events. Our results call for a re-evaluation of what conditions are optimal for coral survival, and the Bonaparte intertidal community presents an ideal model system for exploring how species resilience is conferred in the absence of confounding factors such as pollution.

## Introduction

Reef corals tolerate only a narrow range of environmental conditions; hence widespread coral bleaching events_,_ coupled with land-use impacts, have resulted in rapid and progressive degradation of coral reef habitats [1], [2]. Today, one third of coral species face an elevated risk of extinction [3] and those corals living in intertidal nearshore habitats are particularly threatened [4]. In addition to direct anthropogenic impacts (i.e. habitat modification, pollution, dredging, over-harvesting), intertidal coral communities must withstand multiple abiotic stressors including emersion during low tide, fluctuating temperature, light and wind conditions, physical damage from waves, and sediment and freshwater inundation [5–7]. These impacts may be rapid and pronounced in shallow reef communities [8], [9], hence intertidal fringing reef coral communities are increasingly impoverished [10] and often only the hardiest corals survive in the intertidal zone [11–13].

Scleractinian corals are critical components of the coral reef ecosystem, providing the structural framework of reefs; they contribute to primary production, nutrient recycling, and provide microhabitat and a food source for a wide diversity of coral reef organisms [14]. Hence, resource managers urgently need effective strategies to mitigate the risks imposed on corals to safeguard coral reef ecosystems [15], [16]. One promising approach is to identify existing coral communities that are hardened to climatic extremes and to determine how these communities tolerate stress [17]. To date, only a small number of populations of a restricted subset of species have been shown to tolerate climatic stress (e.g. *Acropora hyacinthus* in Ofu Island Lagoon, American Samoa [18]; back reef communities in the Western Caribbean [19]; and coral communities in the Persian/Arabian Gulf [20]).

In this study we examine the species composition and diversity of reef-building corals growing on intertidal fringing reef flats across three island groups in the Bonaparte Archipelago, Kimberley, north western Australia. We compare the environmental conditions and species diversity of these intertidal communities with those of other shallow fringing reef communities around Australia, and discuss how such a high diversity of coral is sustained in this dynamic and severe environmental setting.

## Methods

### Ethics Statement

All necessary permits were obtained for the described field studies. A coral collection permit was obtained from the Western Australian Fisheries Department, Permit Number—SPA 01/07.

### Study Sites

**Fig 1:**
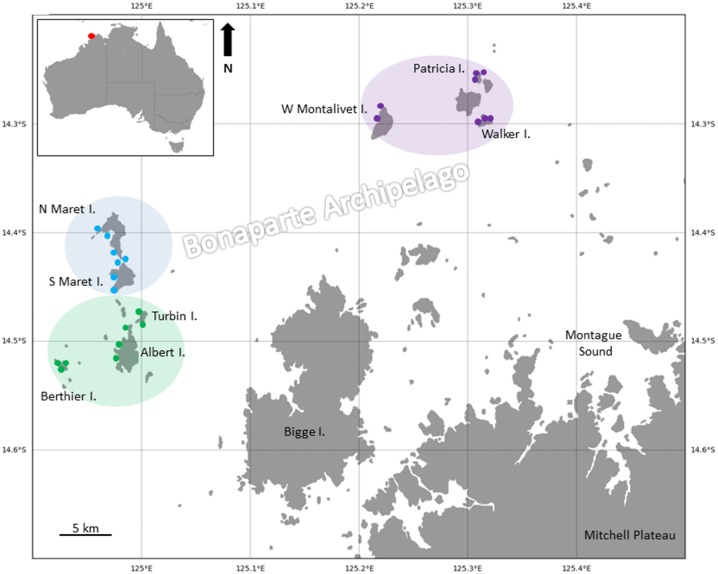
Map of study sites in the Bonaparte Archipelago, Kimberley, Australia. Individual dots indicate survey sites within the three main island groups—Berthier (green), Maret (blue) and Montalivet (purple). Site names and details are listed in Table A of S1 File.

The Bonaparte Archipelago is located in north western Australia (Fig. 1) and is part of the Kimberley Bioregion [21]. The Kimberley consists of many island archipelagos with fringing reefs, platform reefs, submerged banks and offshore atolls [22]. Low energy, macro-tidal conditions characterize the region and the tidally-driven currents together with shelf position and the distance from rivers and estuaries influences the level of turbidity [23].

### Field Surveys

In this study scleractinian coral biodiversity was recorded at 23 sites across ten islands (North and South Maret I., West Montalivet I., East Montalivet I.,Walker I., Patricia I., Berthier I., Albert I., Turbin I., Suffren I.) from three island groups (Maret, Berthier and Montelivet) in the Bonaparte Archipelago (Fig. 1, Table A in S1 File). Saltwater crocodiles (*Crocodylus porosus*) frequent these reefs and diving was prohibited under workplace safety regulations, hence only the intertidal habitat was examined, during low spring tides of 1^st^ September–20^th^ October 2007. Approximately 240 m^2^ of inner and outer reef flat and reef crest habitat was surveyed at each site and all coral species encountered were identified *in situ* or collected for later identification.

Coral diversity was surveyed using a rapid ecological assessment methodology. To determine the relative frequency of species occurrence, all species at each site were classified into one of the following five categories of abundance: Category 1–rare (1–2 colonies); Category 2–infrequent (3–5 colonies); Category 3–frequent (6–20 colonies); Category 4–common (21–50 colonies); and Category 5–dominant (51 or more colonies). Since accurate *in-situ* ID of many coral species is not possible we collected small (5–8cm) skeletal samples which were bleached in a 3% hypochlorite solution overnight and then air-dried and returned to the laboratory for ID. Identifications were carried out with comparison to known and type specimens in the Queensland Museum collection according to: [24] for *Acropora* and *Isopora*; [25] for Fungiidae; [26] for *Psammocora*; [27], [28] for Lobophylliidae, Merulinidae, Montastraeidae and Diploastraeidae; and [29] for all taxa that have not been revised recently. Moreover, the higher-level taxonomic classifications used in this study reflect the classifications listed in the World Register of Marine Species http://www.marinespecies.org/ as of September 2014. New distribution records were verified by discussion with JEN Veron and with comparison to the Corals of the World database: http://www.coralsoftheworld.com. Specimens have been deposited with the Queensland Museum.

### Analyses

To examine the adequacy of local sampling at the Bonaparte Archipelago, a species accumulation curve was calculated using the “specaccum” function of the “vegan” library in R with jack-knifed standard errors http://www.r-project.org/.

To assess the proportion of coral species from our study not normally found in the intertidal zone, we looked for intertidal vs subtidal records for the same species in the Museum of Tropical Queensland database (>28,000 specimen-based records). To compare the diversity observed in the intertidal zone in the Kimberley with that seen in other more typical reef locations, we standardized the area surveyed to 100m^2^ and compared this with a semi-quantitative estimate of the level of diversity recorded elsewhere in the NE Indian Ocean to the NW Pacific Ocean (see Table B in S1 File).

A resemblance matrix based on Bray-Curtis similarities was constructed using square-root transformed abundance data from the 23 sites using PRIMER-E v6 [30]. Agglomerative CLUSTER analysis was used to group the sites according to the similarity in coral assemblage composition using group average linkage distances. We used Krustal’s non-metric multidimensional scaling (MDS) analysis to visualize the variation between sites as a 2-D plot.

### Physical Variables

Hourly tidal predictions for North Maret I. (Bonaparte Archipelago, Kimberley, Australia); (Scott Reef, Offshore Atoll, Kimberley); Barrow I. (Pilbara, Western Australia); Lizard I. (Northern Great Barrier Reef, Australia) and Dent I. (Whitsundays, Central Queensland, Australia) (see Table C in S1 File for co-ordinates) were obtained from the National Tide centre of the Australian Bureau of Meteorology for the years 2002–2014 http://www.bom.gov.au/oceanography/projects/ntc/ntc.shtml. The average proportional occurrence of tidal amplitude per year (2002–2014) at 1 m intervals (± SD) was plotted to compare the distribution of tidal amplitudes between regions. The proportional occurrence of spring low tides (≤2 m) at hourly intervals for North Maret I. was also plotted to illustrate time of day and length of time intertidal corals are exposed to air during different lunar phases (2002–2014).

To provide environmental data we used satellite imagery captured by the MODerate resolution Imaging Spectroradiometer (MODIS) onboard NASA’s Aqua satellite. This sensor captures imagery at many spectral bands from the visible to the far infrared (http://modis.gsfc.nasa.gov/about/) on a near daily repeat cycle. Empirical algorithms are then applied to the spectral information to derive geophysical parameters such as sea surface temperature (SST) [24] and the diffuse down-welling attenuation coefficient {K_d_(490 nm), [31]. We obtained eight day averaged global data at 4 km spatial resolution of SST and K_d_(490 nm) from http://oceandata.sci.gsfc.nasa.gov/MODISA/Mapped/8Day/4km/ from July 2002 to June 2014. The SST data over a 12km × 12km region about a coordinate of 125.0°E/ 14.3750°S were averaged at each time stamp to obtain a SST time series for North Maret I. The same approach was used to obtain average SST estimates for the other four locations and co-ordinates listed in Table C in S1 File.

K_d_(490) is a measure of the light penetrability in the water column and as such is a proxy for turbidity, where higher K_d_(490) values pertains to lesser penetration of light into the water column and hence greater turbidity. The operational MODIS K_d_(490) algorithm relies on a log-transformed ratio between the remote sensing reflectance at 488 and 547 nm and has been validated for oceanic waters with negligible bottom reflectance [32], [33]. Over optically shallow waters such as in coral reefs, the large contribution of bottom reflectance to the above-water radiances can over-estimate parameters that rely on this spectral ratio [34], [35]. To minimize this effect the K_d_(490) values were taken over adjacent deep-water regions for each location and averaged to obtain a time series for each location. These values should therefore be interpreted as the average minimum turbidity at each location. Lastly, we conducted a series of one-way ANOVA’s to test the null hypothesis that there is a significant difference between the SST and K_d_(490) time-series values between North Maret I., and Scott Reef, Barrow I., Lizard I. and Dent I.

## Results and Discussion

### Species Diversity

Based on a skeletal collection of 506 corals from 23 intertidal sites, we document 225 species of hard coral from 60 genera occurring in the northern sector of the Bonaparte Archipelago (Table D in S1 File). Seven of these species are newly recorded from Western Australia (*Goniopora fruitcosa*, *Goniopora norfolkensis*, *Isopora crateriformis*, *Lobophyllia flabelliformis*, *Lobophyllia serratus*, *Platygyra acuta*, *Stylaraea punctata*) and this study extends their distribution range from Indonesia and the NW Pacific to include the eastern Indian Ocean.

Our study provides a robust representation of the observed local species richness (see species accumulation curve—Figure A in S1 File); however this estimate is conservative because we only present data pertaining to species records that have been substantiated with a reference skeletal specimen. Furthermore, intertidal coral communities contain a subset of the local diversity (70–90%) [13], [36] hence we estimate a further 23–68 species could be expected if subtidal habitats were surveyed but further collection efforts are required to verify this.

**Fig 2:**
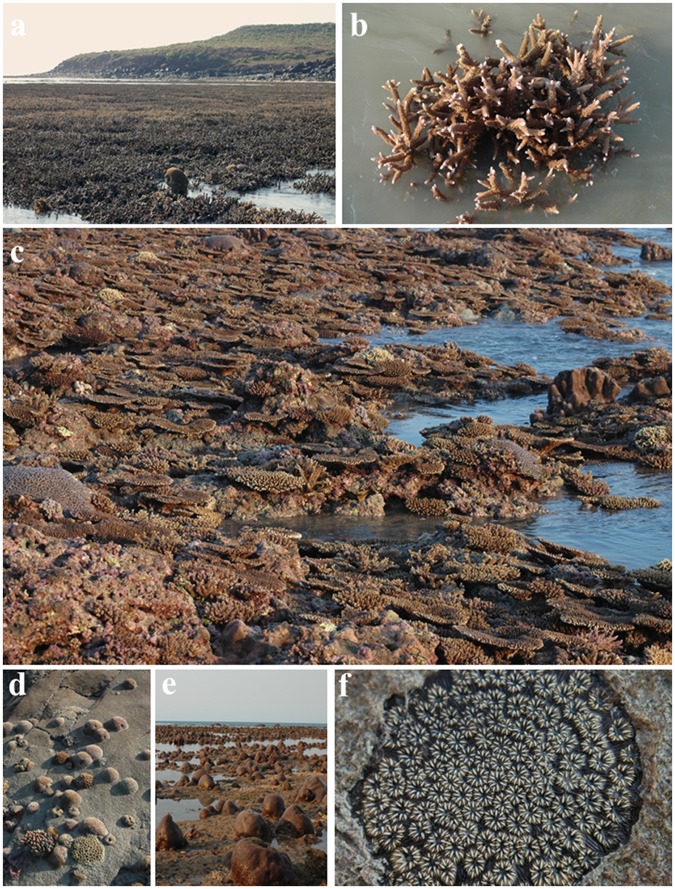
A high diversity of coral thrives in the Bonaparte intertidal zone. (a) Thickets of branching *Acropora aspera* and *A*. *muricata* dominate the inner reef platform at north Patricia I., (b) On low tides corals are exposed to air for up to three and a half hours at a time, (c) A high diversity of *Acropora* species thrive on the outer fringing reef platform at north Patricia I., (d) An aggregation of juvenile and subadult corals inhabiting a steep granite cliff-face on the east side of Walker I., (e) *Goniastrea* coral heads dominate the inner platform at north-west Patricia I.,(f). *Oulastrea crispata* a rare and distinctive species that normally occurs subtidally was encrusting a granite boulder on the rocky shore of Walker I.

By comparing the current intertidal records with over 28,000 specimen-based depth distribution records in the Museum of Tropical Queensland we document 34 species in the intertidal zone that have previously been recorded only from subtidal habitats (e. g. *Echinopora gemmacea*, *Stylaraea punctata*, *Oulastrea crispata—* Fig. 2f) (Table D in S1 File). Numerous other species we recorded in this inshore habitat were only previously recorded from offshore clear water habitats (e.g. *Leptastrea pruinosa*, *Astreopora myriophthalma* [29].

The diversity of hard corals in the three island groups of the Bonaparte Archipelago was similar to, or higher than a semi-quantitative estimate of the level of diversity in intertidal or shallow subtidal habitats (0–5 m depth) in other parts of the Indo-Pacific (Figure B in S1 File). The level of diversity was similar to that estimated for inshore fringing reefs in the central sector of the Great Barrier Reef (i.e. Dent I., Border I., Whitsundays) ~ 2 decades ago [37] prior to the well documented decline of coral cover and condition of the inshore mid to southern sections of the Great Barrier Reef [38–40].

**Fig 3:**
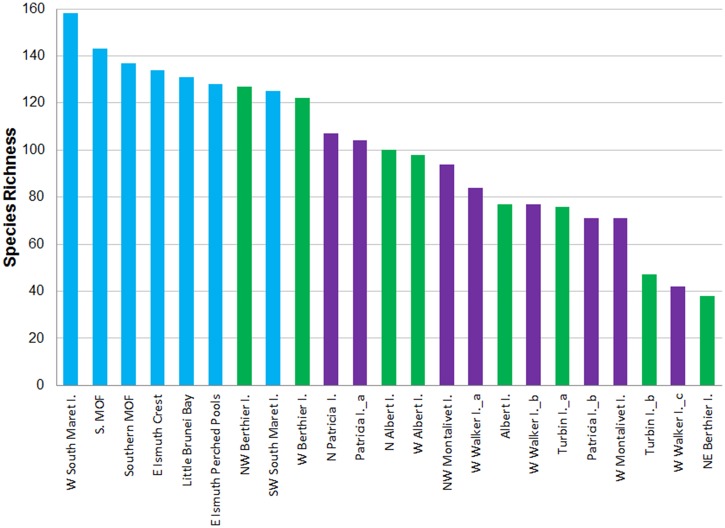
Spatial variation in the total species richness recorded at 23 sites spanning three island groups in the Northern sector of the Bonaparte Archipelago. Maret Is. group (blue columns); Berthier Is. group (green columns) and Montalivet Is. group (purple columns).

**Fig 4:**
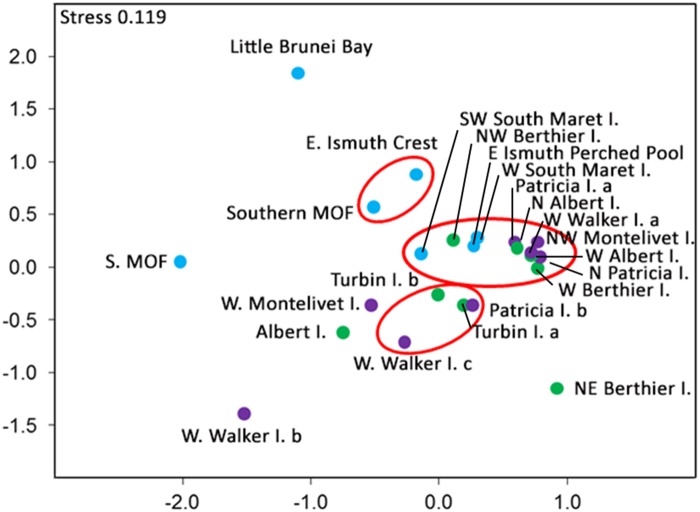
Spatial variation in the composition of the coral assemblages at three island groups in the Bonaparte Archipelago. Maret Group (blue dots); Berthier Group (green dots) and Montalivet Group (purple dots). Kruskal’s non-metric multidimensional scaling (nm-MDS), using Bray-Curtis similarity index of the coral assemblage at 23 sites based on relative abundance data. Linkages are based on weighted pair group averages and ellipses indicate those sites with 60% similarity at P < 0.001.

Species richness was highest within the Maret group and peaked on the western side of South Maret I. (n = 158) (Fig. 3). All Maret I. sites had over 120 species. The diversity of corals at sites in the Berthier group ranged from 38–127 species, while diversity of corals at sites within the Montalivet group ranged from 42–107 species. The spatial variation in the composition of coral assemblages is clear in the 2D nmMDS plot which shows Little Brunei Bay and S. MOF sites at Maret I. being distinctive from other sites in Maret I. (Fig. 4). There is a degree of overlap in the coral assemblages across the three island groups, with 11 of the sites grouping together with 60% similarity.

Seven species were locally widespread (*Pocillopora damicornis*; *Symphyllia recta*; *Acropora hyacinthus*, *Galaxea astreata*, *Coelastrea aspera*, *Lobophyllia hemprichii and Stylophora pistillata*) and 33 other species, including five *Acropora* species (*A*. *aspera*, *A*. *millepora*, *A*. *intermedia*, *A*. *muricata* and *A*. *valida*), were present at more than ¾ of sites surveyed. There were many rare species, seventeen of which were recorded at a single site only. One of these, *Lobophyllia serratus*, is listed as *Endangered* in the IUCN Red List of Threatened Species (www.iucn.redlist.org), and *Stylaraea punctata* is listed as *Data Deficient*.

The distinctiveness of the Bonaparte intertidal coral assemblage is exemplified by the high diversity of *Acropora* species living there (47 spp.) (Figure C in S1 File). *Acropora* are one of the most thermally sensitive and threatened coral genera [3], and it is increasingly rare to find diverse and abundant assemblage of *Acropora* on intertidal nearshore fringing reefs. On the Great Barrier Reef for example, nearshore reefs have been classified as “non-*Acropora* reefs” [41] due to the relative paucity of *Acropora* spp. Not only was *Acropora* the most diverse of the 60 genera of scleractinian coral recorded (Figure C in S1 File), but six *Acropora* species dominated the community (Fig. 2a-c). An abundance of *Acropora* spp. has been reported from other intertidal locations in the Kimberley (e.g. Turtle Reef at Talbot Bay and One-Arm Point, Cape Leveque, in the Western Kimberley [22]) indicating this region may provide a critical refuge for this increasingly threatened group of corals.

### Physical Variables

**Fig 5:**
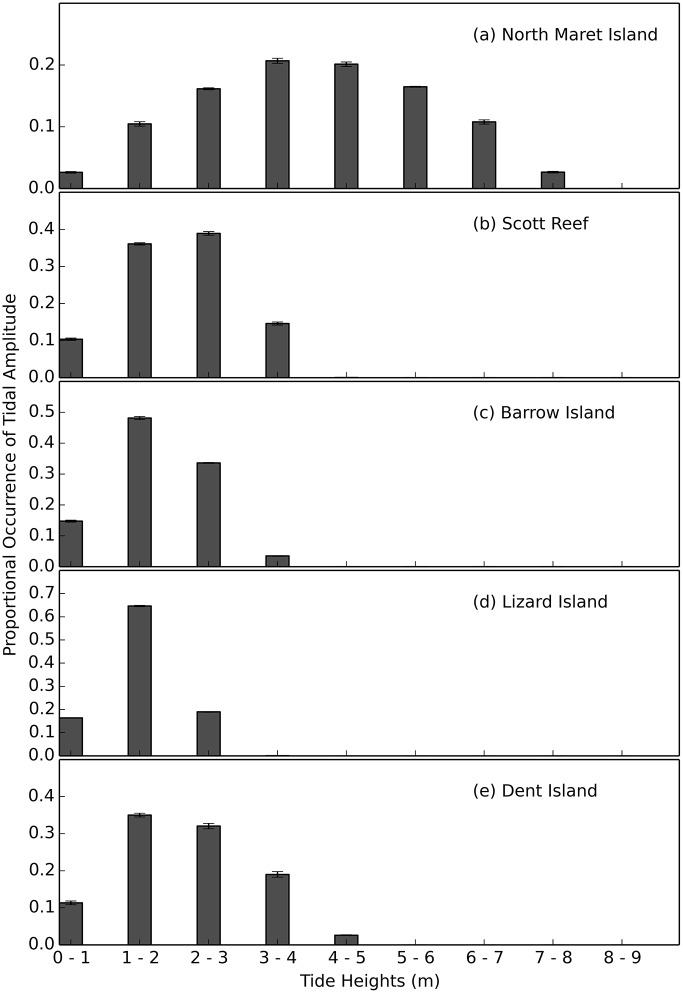
Spatial comparison of tidal amplitude at 1 m intervals (± SD) from 2002–2014. Presented is the proportional occurrence of tide heights for (a) North Maret I., (b) Scott Reef (c) Barrow I., (d) Lizard I, and (e) Dent Island.

**Fig 6:**
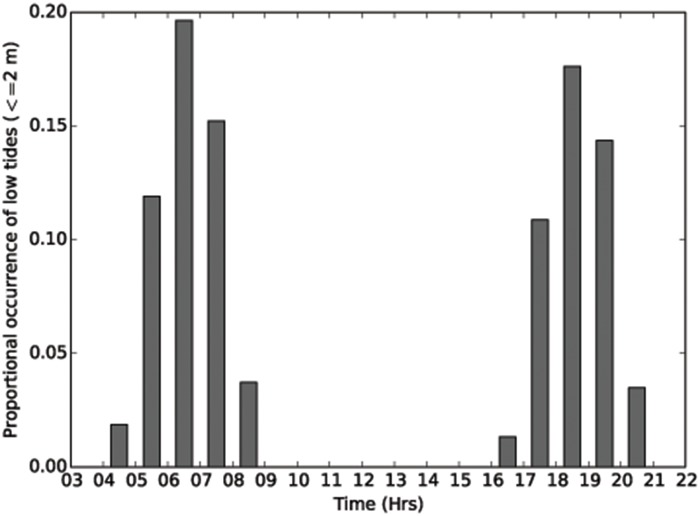
Hourly tide height data of spring low tides for North Maret I. from 2002–2014. The lengths of time corals are exposed for each day depends upon the lunar phase (see Figure D in S1 File) and maximum low tide exposure occurs in early morning and evening.

Hourly tide height data from North Maret I., shows the semidiurnal patterns of the tides which oscillate up to 8m over spring tides (Fig. 5a). When the mean proportional occurrence of tidal heights for North Maret I. is contrasted with four other more typical reef locations (Fig. 5b-e) it is evident tidal conditions in the vicinity of North Maret I. are more dynamic and reach amplitudes up to 3m greater than those in the other more typical reef locations with a similar level of diversity. During spring low tides (i.e. tides ≤ 2m), corals growing on the intertidal reef platform at North Maret I. are exposed to the air for up to 3.5 hours at a time (Figure D in S1 File). However, an important physical feature of the Bonaparte Archipelago is that the spring low tides occur in the early morning, 4am-9am, and late afternoon to early evening, 4pm–9pm (Fig. 6). Thus, corals remain submerged over the hottest parts of the day and are, buffered from the stresses arising from subaerial exposure. Nevertheless, even when corals are submerged, other environmental factors come into play such as sea-surface temperatures and turbidity.

**Fig 7:**
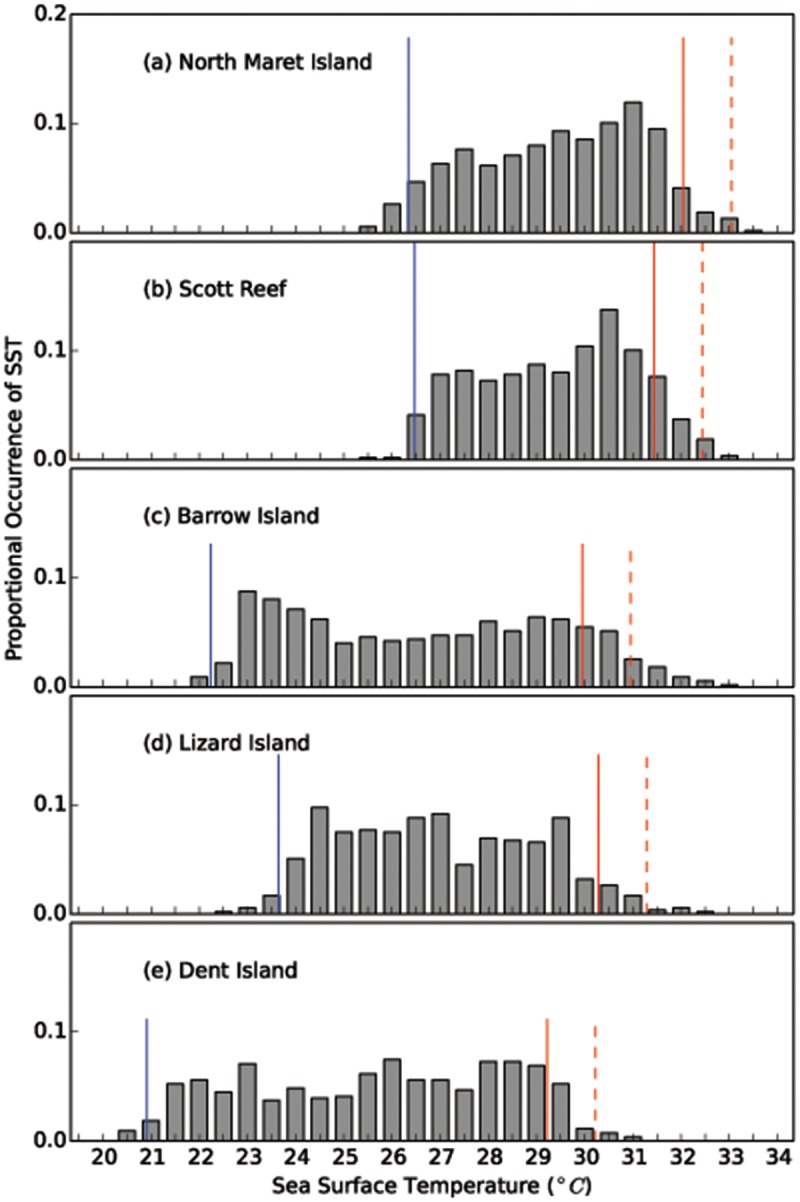
Spatial comparison of sea-surface temperatures from 2002–2014. Presented is the proportional occurrence of SST (a) North Maret I., (b) Scott Reef (c) Barrow I., (d) Lizard I, and (e) Dent Island. The red line shows the mean max. summer SST; and the red dashed line (- - -) shows the + 1°C bleaching threshold (*sensu* NOAA); the blue line shows the mean min. winter SST.

Eight-day average MODIS-derived SST from July 2002 to June 2014 shows that the average SST at North Maret I. ranged from 25.2 to 34.3°C (Table 1). The maximum mean summer SST over this period was 32.2 ± 1.0°C and the minimum mean winter SST was 26.5 ± 1.0°C encompassing a range of 5.7°C. The +1°C bleaching threshold (*sensu* NOAA Coral Reef Watch methods—see http://coralreefwatch.noaa.gov/satellite/index.php) at North Maret I. is 33.2°C and our data suggest SST remained above this threshold for two 8-day periods in January and February 2013 (Figure E in S1 File). When compared with more typical reef locations (Fig. 7) the mean SST in the vicinity of North Maret I. is significantly higher than Lizard I., Barrow I. and Dent I. but not significantly different from Scott Reef (Table E in S1 File). While Scott Reef has succumbed to bleaching events in the past [43], to date there is no evidence to suggest the intertidal coral communities in the Bonaparte Archipelago have experienced a bleaching event (despite NOAA issuing numerous bleaching alerts e.g Mar-Jun 2013). Even though this region is remote, the Bonaparte Archipelago is intermittently visited by scientists (WA Museum, Australian Institute of Marine Science, Department of Fisheries; Cygnet Bay Research Station); tourist vessels and the Australian customs service provide surveillance. While widespread bleaching was been reported across almost 2000 km of Western Australian coastline during the summer of 2010/11 [42] there is no suggestion that the inshore Kimberley reefs have experienced a widespread bleaching event to date.

**Table 1:** Summary statistics for SST parameters at North Maret I. in comparison to three other more typical coral reef locations (unit = °C).

	North Maret I.	Scott Reef	Barrow I.	Lizard I.	Dent I.
**Minimum SST**	25.193	25.120	21.714	22.147	20.040
**Maximum SST**	34.350	32.747	32.669	32.013	30.936
**Average SST**	29.216	29.170	26.439	26.743	25.414
**Standard Deviation SST**	1.790	1.623	2.733	2.036	2.619
**Max. Summer SST mean**	32.208	31.607	30.114	30.450	29.384
**Max. Summer SST Standard Deviation**	0.978	0.692	1.302	0.817	0.732
**Summer SST mean**	30.389	30.225	28.570	29.124	28.401
**Summer SST Standard Deviation**	1.328	1.113	1.458	0.925	0.941
**Min Winter SST mean**	26.504	26.635	22.407	23.808	21.077
**Min Winter SST Standard Deviation**	0.962	0.751	0.615	0.789	0.772
**Winter SST mean**	27.128	27.283	23.325	24.460	22.110
**Winter SST Standard Deviation**	0.989	0.782	0.896	0.792	1.005

Data relate to 8-day global averages derived from the MODIS aqua satellite at a 4-km spatial resolution from July 2002 to June 2014. See Table C in S1 File for site co-ordinates.

The eight-day average of K_d_(490) ranged from 0.03–0.18 m^-1^ at North Maret I. (Table 2). The K_d_(490) values represent natural turbidity levels at North Maret Island. They are slightly lower than the values detected at Barrow I. (Pilbara), a location that has been exposed to a large multi-year dredging operation [44], [45] (Figure F in S1 File) and not significantly different from Dent Island (inshore Whitsundays, GBR) (Table E in S1 File). There are seasonal differences in the K_d_(490) values obtained across all locations with the highest turbidity recorded in Winter at North Maret I. (Figure G in S1 File). It is likely the similarity of summer K_d_(490) values across the locations (Table 2) reflects the effects of tropical cyclones and monsoonal storms on water quality.

**Table 2:** Summary statistics K_d_(490) parameters at North Maret I. in comparison to three other more typical coral reef locations (unit = m^-1^).

	North Maret I.	Scott Reef	Barrow I.	Lizard I.	Dent I.
**Minimum K**_**d**_ **(490)**	0.032	0.019	0.048	0.019	0.029
**Maximum K**_**d**_ **(490)**	0.178	0.117	0.184	0.091	0.174
**Average K**_**d**_ **(490)**	0.063	0.032	0.079	0.046	0.060
**Standard Deviation K**_**d**_ **(490)**	0.017	0.009	0.023	0.011	0.015
**Winter-time K**_**d**_ **(490) mean**	0.068	0.040	0.073	0.044	0.059
**Winter-time K**_**d**_ **(490) Standard Deviation**	0.010	0.007	0.021	0.008	0.010
**Summer-time K**_**d**_ **(490) mean**	0.061	0.029	0.086	0.048	0.060
**Summer-time K**_**d**_ **(490) Standard Deviation**	0.021	0.006	0.023	0.012	0.017
**Autumn-time K**_**d**_ **(490) mean**	0.068	0.030	0.094	0.050	0.072
**Autumn-time K**_**d**_ **(490) Standard Deviation**	0.021	0.009	0.024	0.050	0.015
**Spring-time K**_**d**_ **(490) mean**	0.055	0.027	0.065	0.043	0.052
**Spring-time K**_**d**_ **(490) Standard Deviation**	0.012	0.004	0.009	0.007	0.009

Data relate to 8-day global averages derived from the MODIS aqua satellite at a 4-km spatial resolution from July 2002 to June 2014. See Table C in S1 File for site details.

### Factors Driving Diversity

The findings of our preliminary comparison of regional diversity suggest that the level of coral diversity in the Bonaparte intertidal zone is roughly equivalent to, or greater than that documented from other more typical shallow-water reefs in the NE Indian Ocean and Western Pacific Ocean. It is important to note however that the environmental conditions in the Bonaparte Archipelago are far more extreme and dynamic than these other locations. Hence the question must be asked, how is the diversity of the Bonaparte community being sustained when intertidal coral communities all around the world are becoming increasingly impoverished due to coral bleaching and sediment impacts?

There are a number of possible explanations for the high diversity. Firstly, the Bonaparte Archipelago occurs at low latitude (~14° 24′ S) and in relatively close proximity to Indonesia (~500 km) where the greatest level of reef coral diversity is documented [46]. While it is well known that coral diversity increases towards the equator [47], [48] the diversity of corals occurring in the Kimberley region has been under-represented in studies of coral biogeography and biodiversity [49]. Hence the extent of faunal connectivity between Indonesia and NW Australia has not been quantified. The Timor and Banda Seas were continuously connected over the Quaternary via the Indonesian Throughflow Current [22] and this, coupled with our finding of a degree of affinity between the Kimberley and Indonesian coral faunas, supports the premise that the diversity of the Kimberley coral fauna is at least partly sustained through connections with Indonesia.

Secondly, there is a strong link between declining water quality, proximity to urban centres and the condition of coral reefs [50–52], hence we postulate that a low level of pollution and development helps to explain how this remarkable diversity of coral is sustained. The Kimberley region is sparsely populated with no major urban centres. In 2011 just over 34,000 people lived in the region (423,517 km²) (http://kdc.wa.gov.au/Statistics/Census-Profiles), compared to 1.1 million people living in the Great Barrier Reef catchment area (425 964 km^2^) in the same year [53]. The Kimberley has a large pastoral industry but only a relatively small area of land that is irrigated or used for horticulture (http://kdc.wa.gov.au/economic-activity/agriculture). Thus, in contrast to the east coast of Australia and in many other parts of the world [2], [54–56] there are no major urban centres in the Kimberley, and the input of agricultural-based nutrients and pesticides into the nearshore marine ecosystem is minimal.

Another factor likely to help explain how coral diversity is sustained is the distinctive tidal regime. The geographic position and shelf bathymetry of the Kimberley have resulted in the region being characterized by tides that reach their maximum spring and summer amplitudes in the early morning and late afternoon/evening (Fig. 6, Figure D in S1 File). This means that the intertidal corals are in effect, protected from subaerial emersion and desiccation-based stresses over the hottest parts of the day (10am-3pm). Furthermore, the apparent ability of corals to occupy a broad physiological niche may also relate to other environmental variables such as cloud cover, turbidity and the strong currents [57], [58].

The macro-tidal conditions dictate that the inshore Kimberley region is dynamic and even in the absence of flood or storm events, the large tides and subsequent currents create “water boils” during spring tides, when fine sediments on the seafloor are resuspended [22]. Thus, unlike the clear waters that characterize most coral reef ecosystems, this inshore Kimberley is characterized by turbid water. Ordinarily, high levels of suspended sediment are thought to restrict light availability and prevent the settlement and colonization of coral larvae [63]. However in the Kimberley, we postulate that the naturally high levels of suspended sediments may actually protect the shallow water corals from solar radiation [59] by back-scattering light and lowering the intensity of down-welling irradiance reaching the benthos [60], [61].

While tidal-driven cyclical variation in benthic irradiance could result in corals fluctuating between states of potential light limitation, to light stress [62]; overall, the Bonaparte corals maintain a positive energy balance. Thus in this system, the expected negative effects of heat, light and sediment may be transient and/or accommodated due to the large tides and strong currents which provide water movement and aeration. The Kimberley corals are also likely have adapted to the local conditions by the enhanced production of mucus and tissue inflation [64] and/or increased levels of heterotrophic feeding [65]; or via other physiological adaptations (see [66–69] for examples) however the traits that underpin survival remain to be explored.

## Conclusion

Here we report the finding of an exceptionally diverse intertidal fringing reef community in the Kimberly region of north-western Australia, which thrives despite extreme environmental controls. The presence of this diverse community calls for a re-evaluation of what conditions are optimal for coral survival. Our results may elicit some optimism about the future of corals reefs because we demonstrate that in the absence of additional stressors, diverse assemblages of coral can thrive in atypical and dynamic environmental settings. The assemblage we report here provides an indication of which corals may have existed in other nearshore locations in the past and presents an ideal model system for exploring how resistance and resilience are conferred in the absence of confounding factors such as pollution. In the future, genetic material from the hardy Kimberley corals may help to boost the resilience of corals in other parts of the world (i.e. through natural gene flow or genetic translocation and preservation [17], [70]) and may circumvent the need to artificially design ‘smart reefs’ [71]. Nevertheless, the Kimberley coral communities are by no means immune from climatically or anthropogenically-imposed changes, hence ongoing local resource management and conservation action are vital.

## Supporting Information

S1 File Combined supporting information file. **Table A.** Details of the 23 study sites in the Bonaparte Archipelago. **Table B.** Location, co-ordinates, method and approximate area surveyed of our study sites and additional sites used for comparative purposes. **Table C.** Co-ordinates for physical variables. **Table D.** Annotated species list. Listed are the specimen accession numbers and site occupancy at local, group and regional scales including known depth zone i.e. <5m (intertidal) or >5m (subtidal) based upon the specimen-based records in the Queensland Museum coral database. **Table E.** Summary of significance results from one-way analysis of variance comparing the mean SST and Kd(490) time-series data between locations. **Figure A.** Permutated species accumulation curves. The local species diversity was adequately surveyed after approximately 20 sites were surveyed in the Bonaparte Archipelago. **Figure B.** Semi-quantitative spatial comparison of coral species diversity. This figure illustrates that the three Bonaparte Island groups (Maret I., Berthier I., and Montalivet I., *in blue*) have a similar level of diversity to that estimated for other more typical and less physically extreme reef locations such as Dent I. and Border I. on the Great Barrier Reef and Christmas I., an offshore oceanic location in the NE Indian Ocean. The level of diversity per 100m^2^ is higher than Ashmore Reef (Offshore Kimberley); Lizard I. (Northern GBR); Kosrae and Maju ro Atoll (Central Pacific) and the Red Sea. Data summarized from [37], [72–77]. **Figure C.** Species level diversity within genera at the 23 intertidal survey sites. Note: 27 genera were represented by a single species (Table D in S1 File). **Figure D.** Daily tidal cycle at North Maret Island on selected spring and neap tides over our survey period in October 2007. During spring low tides (i.e. tides ≤ 2m), corals growing on the intertidal reef platform at North Maret I. are exposed to the air for up to 3.5 hours at a time whereas at neap tide, corals remain submerged by at least 1m of water. See Fig. 5 for a time-series analysis showing the proportion of tides occurring at 1m intervals from 0–1m up to 7–8m. **Figure E.** Time series (2002–2014) showing SST data based on 8-day averages for 5 locations. This figure shows at North Maret I. SST surpassed the +1°C bleaching threshold in Feb-March 2013. **Figure F**. Spatial comparison of K_d_(490), 2002–2014. K_d_(490) represents the diffuse attenuation coefficient of down-welling irradiance at 490 nm and is used as a measure of turbidity. **Figure G.** K_d_(490) time series for North Maret I. from 2002–2014. The blue line represents the average winter turbidity level and the red represents the average summer turbidity.
(DOC)
