# Supplementary material for: A Diverse Assemblage of Reef Corals Thriving in a Dynamic Intertidal Reef Setting (Bonaparte Archipelago, Kimberley, Australia)
Source: PLoS One. 2015 Feb 25;10(2):e0117791. doi: 10.1371/journal.pone.0117791 (PMC4340616; doi:10.1371/journal.pone.0117791)
Supplement: S1 File — Table A. Details of the 23 study sites in the Bonaparte Archipelago. Table B. Location, co-ordinates, method and approximate area surveyed of our study sites and additional sites used for comparative purposes. Table C. Co-ordinates for physical variables. Table D. Annotated species list. Listed are the specimen accession numbers and site occupancy at local, group and regional scales including known depth zone i.e. <5m (intertidal) or >5m (subtidal) based upon the specimen-based records in the Queensland Museum coral database. Table E. Summary of significance results from one-way analysis of variance comparing the mean SST and Kd(490) time-series data between locations. Figure A. Permutated species accumulation curves. The local species diversity was adequately surveyed after approximately 20 sites were surveyed in the Bonaparte Archipelago. Figure B. Semi-quantitative spatial comparison of coral species diversity. This figure illustrates that the three Bonaparte Island groups (Maret I., Berthier I., and Montalivet I., in blue) have a similar level of diversity to that estimated for other more typical and less physically extreme reef locations such as Dent I. and Border I. on the Great Barrier Reef and Christmas I., an offshore oceanic location in the NE Indian Ocean. The level of diversity per 100m2 is higher than Ashmore Reef (Offshore Kimberley); Lizard I. (Northern GBR); Kosrae and Maju ro Atoll (Central Pacific) and the Red Sea. Data summarized from [37], [72–77]. Figure C. Species level diversity within genera at the 23 intertidal survey sites. Note: 27 genera were represented by a single species (Table D in S1 File). Figure D. Daily tidal cycle at North Maret Island on selected spring and neap tides over our survey period in October 2007. During spring low tides (i.e. tides ≤ 2m), corals growing on the intertidal reef platform at North Maret I. are exposed to the air for up to 3.5 hours at a time whereas at neap tide, corals remain submerged by at least 1m of [file pone.0117791.s001.doc]

Supporting Information S1 File for:

**A diverse assemblage of reef corals thriving in a dynamic reef setting (Bonaparte Archipelago, Kimberley, Australia).**

Richards, ZT^1^, Garcia RA^2^, Wallace CC^3^, Rosser NL^4^, Muir PR^3^

*To whom correspondence should be addressed to:

Zoe Richards: Tel: 61-8-92123872; Fax: 61-8-92123882; email: [zoe.richards@museum.wa.gov.au](mailto:zoe.richards@museum.wa.gov.au)

**File S1 includes:**

Tables A - E

Figures A - G

**Table A. Details of the 23 coral diversity study sites in the Bonaparte Archipelago.**

| **Island Group** | **Island** | **Site Details - Exposure** | **Date** | **Latitude (South)** | **Longitude (East)** |
| --- | --- | --- | --- | --- | --- |
| Berthier | Albert Island | West | 29/10/2007 | 14° 32' 26" | 124° 55' 42" |
| Berthier | Albert Island | North | 30/10/2007 | 14° 31' 27" | 124° 55' 41" |
| Berthier | Berthier Island | North-east | 27/10/2007 | 14° 30' 30" | 124° 58' 36" |
| Berthier | Berthier Island | North-west | 28/10/2007 | 14° 29' 46" | 124° 59' 16" |
| Berthier | Berthier Island | West | 27/10/2007 | 14° 30' 26" | 124° 58' 24" |
| Montalivet | East Montalivet Island |  | 27/09/2007 | 14° 17' 28" | 125° 13' 3" |
| Maret | North Maret Island | E Ismuth crest | 28/09/2007 | 14° 24' 46" | 124° 58' 20" |
| Maret | North Maret Island | E Ismuth Perched Pools | 28/09/2007 | 14° 26' 51" | 124° 58' 37" |
| Maret | North Maret Island | Little Brunei Bay | 29/09/2007 | 14° 24' 02" | 124° 58' 02" |
| Montalivet | Patricia Island | North | 29/09/2007 | 14° 15' 33" | 125° 18' 27" |
| Montalivet | Patricia Island | _a North-west | 28/09/2007 | 14° 15' 29" | 125° 18' 25" |
| Montalivet | Patricia Island | _b | 27/09/2007 | 14° 18' 28" | 125° 18' 3" |
| Maret | South Maret Island | S. MOF | 27/09/2007 | 14° 24' 46" | 124° 58' 20" |
| Maret | South Maret Island | South-west | 28/09/2007 | 14° 26' 51" | 124° 58' 37" |
| Maret | South Maret Island | West | 30/09/2007 | 14° 26' 46" | 124° 58' 40" |
| Maret | South Maret Island | Southern MOF | 1/10/2007 | 14° 26' 23" | 124° 58' 28" |
| Berthier | Suffren Island |  | 29/10/2007 | 14° 32' 18" | 124° 55' 30" |
| Berthier | Turbin Island | _a | 28/10/2007 | 14° 28' 30" | 124° 59' 30" |
| Berthier | Turbin Island | _b | 30/10/2007 | 14° 29' 0" | 124° 59' 36" |
| Montalivet | Walker Island | West_a | 1/09/2007 | 14° 15' 44" | 125° 18' 43" |
| Montalivet | Walker Island | North | 1/09/2007 | 14° 17' 55" | 125° 16' 1" |
| Montalivet | Walker Island | West_b | 30/09/2007 | 14° 15' 44" | 125° 18' 43" |
| Montalivet | West Montalivet Island |  | 28/09/2007 | 14° 17' 28" | 125° 13' 3" |

**Table B.** **Location, co-ordinates, method and approximate area surveyed of our study sites and additional sites used for comparative purposes**.

| **Location** | **Region** | **Country** | **Latitude** | **Longitude** | **Method** | **Approximate area surveyed** | **Source** |
| --- | --- | --- | --- | --- | --- | --- | --- |
| Berthier Group | Kimberley | Australia | S 14° 31.017299' | E 124° 58.951721' | Rapid visual assessment _ intertidal zone | 1920 m^2^ | This study |
| Maret Group | Kimberley | Australia | S 14° 24.065803' | E 124° 58.498535' | Rapid visual assessment _intertidal zone | 1680 m^2^ | This study |
| Montalivet Group | Kimberley | Australia | S 14° 18.358912' | E 125° 13.610101' | Rapid visual assessment _intertidal zone | 1920 m^2^ | This study |
| Lizard Island | Northern Great Barrier Reef | Australia | S 14° 40.974429' | E 145° 27.600861' | 3 replicate 50m x 2m belt transects _ 3-5m depth | 4200 m^2^ | Richards 2013a |
| Christmas Island | Australia | Australia | S10° 25.784488' | E105° 40.039158' | 3 replicate 15m x 2m belt transects _ 5m depth | 720 m^2^ | Ryan, Richards & Hobbs 2014 |
| Kosrae | Caroline Islands | Federated States of Micronesia | N 5° 15.632273' | E 162° 59.224205' | 3 replicate 50m x 2m belt transects _ 3-5m depth | 1500 m^2^ | Richards 2013b |
| Ashmore Reef | Timor Sea | Australia | S 12° 20.606554’ | E 122° 99.998801’ | 3 replicate 50m x 2m belt transects _3-5m depth | 1800 m^2^ | Richards et al., 2009 |
| Majuro | Marshall Islands | Republic of the Marshall Is. | N 7° 07.198253' | E 171° 11.173096' | 3 replicate 50m x 2m belt transects _ 3-5m depth | 3900 m^2^ | Richards and Beger, 2011 |
| Dent Island | Central Great Barrier Reef | Australia | S 20° 20.948014' | E 148° 55.675049' | Rapid visual assessment _ 0-3m depth | 600 m^2^ | Devantier et al., 1998 |
| Pelican Island | Central Great Barrier Reef | Australia | S 20° 20.171808' | E 148° 51.32472' | Rapid visual assessment _ 0-3m depth | 600 m^2^ | Devantier et al., 1998 |
| Border Island | Central Great Barrier Reef | Australia | S 20° 10.02932' | E 149° 01.659164' | Rapid visual assessment _ 0-3m depth | 600 m^2^ | Devantier et al., 1998 |
| Eliat | Gulf of Aqaba | Red Sea | N 29° 31.39683' | E 34° 56.191406' | 84 replicate 10m long transects _ 0-5m depth | 840 m^2^ | Loya, 1972 |

**Table C.** Co-ordinates for physical variables.

| Location | ​Latitude SST | Longitude SST | Kd490 details |
| --- | --- | --- | --- |
| North Maret Island, Kimberley | 14.3750° S | 125.0000° E | Deep water pixels to the north and west of North Maret I. |
| Dent Island, Whitsundays, GBR | 20.3500° S | 148.9333° E | Deep water pixels from north east to south east of Whitsunday I. |
| Lizard Island, Nth GBR | 14.6666° S | 145.4583° E | Deep water pixels to the north, east and south of Lizard I. |
| Scott Reef, NW, WA | 14.0833° S | 122.1250° E | Grid in the lagoon or on the eastern side of south reef |
| Barrow Island, Pilbara,WA | 20.7917° S | 115.7083° E | Grid from Eastern Side of the Island |

**Table D. Annotated species list**. Listed are the specimen accession numbers and site occupancy at local, group and regional scales including known depth zone i.e. <5m (intertidal) or >5m (subtidal) based upon the specimen-based records in the Queensland Museum coral database.

| Family | Species | MTQ Registration Number | Berthier Island Group (s=8) | Maret Island Group (s=7) | Montalivet Island Group (s=8) | Total Sites Occupied (s=23) | Intertidal or Subtidal in QM database |
| --- | --- | --- | --- | --- | --- | --- | --- |
| Acroporidae | *Acropora aculeus* (Dana, 1846) | G64398 | - | 5 | - | 5 | Intertidal |
| Acroporidae | *Acropora acuminata* (Verrill, 1864) | G61084 G61663 G61683 | 1 | - | 4 | 5 | Intertidal |
| Acroporidae | *Acropora anthocercis* (Brook, 1893) |  | 1 | - | 2 | 3 | Intertidal |
| Acroporidae | *Acropora arafura* Wallace, Done and Muir | G61675 G61684 G63138 G63140 G63141 G63142 G63143 G63144 G63145 G63236 G64399 | 2 | 5 | 2 | 9 | Intertidal |
| Acroporidae | *Acropora aspera* (Dana, 1846) | G60642 G61665 G61673 | 7 | 7 | 7 | 21 | Intertidal |
| Acroporidae | *Acropora austera* (Dana, 1846) | G61081 G64339 | 5 | - | 5 | 10 | Intertidal |
| Acroporidae | *Acropora cerealis* (Dana, 1846) | G60657 G61088 G61584 G61686 | 3 | 7 | 4 | 14 | Intertidal |
| Acroporidae | *Acropora clathrata* (Brook, 1891) | G60662 G61672 G64337 | 4 | 7 | 7 | 18 | Intertidal |
| Acroporidae | *Acropora cytherea* (Dana, 1846) |  | - | 7 | 5 | 12 | Intertidal |
| Acroporidae | *Acropora digitifera* (Dana, 1846) | G61670 G61676 G60623 G60639 G60646 G60647 G60652 G61089 G61090 | 8 | 7 | 4 | 19 | Intertidal |
| Acroporidae | *Acropora divaricata* (Dana, 1846) |  | - | 3 | 3 | 6 | Intertidal |
| Acroporidae | *Acropora florida* (Dana, 1846) | G61092 | 6 | 5 | 5 | 16 | Intertidal |
| Acroporidae | *Acropora gemmifera* (Brook, 1892) | G60620 G60626 G60654 | 5 | 4 | 5 | 14 | Intertidal |
| Acroporidae | *Acropora glauca* (Brook, 1893) | G60631 G60635 G60641 G60664 G61678 G61688 G61690 G64293 G64386 G64391 G64396 G64397 | 2 | 1 | 3 | 6 | Intertidal |
| Acroporidae | *Acropora grandis* (Brook, 1892) |  | - | 5 | 2 | 7 | Intertidal |
| Acroporidae | *Acropora humilis* (Dana, 1846) | G61585 G64390 | 6 | 6 | 6 | 18 | Intertidal |
| Acroporidae | *Acropora hyacinthus* (Dana, 1846) | G60632 G60661 G60665 G61091 G61661 G64395 | 8 | 7 | 7 | 22 | Intertidal |
| Acroporidae | *Acropora intermedia* (Brook, 1891) | G60628 G60633 G60643 G61689 | 6 | 7 | 7 | 20 | Intertidal |
| Acroporidae | *Acropora latistella* (Brook, 1891) | G60619 G60656 G61093 G61094 G61680 | 4 | 3 | 4 | 11 | Intertidal |
| Acroporidae | *Acropora listeri* (Brook, 1893) | G64307 | - | 1 | - | 1 | Intertidal |
| Acroporidae | *Acropora loripes* (Brook, 1892) |  | 4 | - | 3 | 7 | Intertidal |
| Acroporidae | *Acropora lutkeni* Crossland, 1952 | G60660 G61681 | 1 | - | 3 | 4 | Intertidal |
| Acroporidae | *Acropora microclados* (Ehrenberg, 1834) |  | - | 6 | - | 6 | Intertidal |
| Acroporidae | *Acropora microphthalma* (Verrill, 1859) | G64336 | 2 | 7 | 5 | 14 | Intertidal |
| Acroporidae | *Acropora millepora* (Ehrenberg, 1834) | G61087 | 6 | 7 | 7 | 20 | Intertidal |
| Acroporidae | *Acropora monticulosa* (Brüggemann, 1879) |  | - | - | 3 | 3 | Intertidal |
| Acroporidae | *Acropora muricata* (Linnaeus, 1758) | G61068 G61069 G61070 G61095 G61256 G61257 G61258 G61259 G61260 G61261 G61262 G61586 G61667 G61685 G61691 G61692 G61693 | 7 | 7 | 7 | 21 | Intertidal |
| Acroporidae | *Acropora nana* (Studer, 1878) | G61085 G61086 G61587 | 4 | 6 | 1 | 11 | Intertidal |
| Acroporidae | *Acropora nasuta* (Dana, 1846) | G60622 G60627 G60638 G60648 G60649 G60650 G60663 G61664 G61671 | 5 | 6 | 7 | 18 | Intertidal |
| Acroporidae | *Acropora palmerae* Wells, 1954 | G64322 | 1 | 5 | - | 6 | Intertidal |
| Acroporidae | *Acropora papillare* Latypov, 1992 |  | 1 | 1 | 4 | 6 | Intertidal |
| Acroporidae | *Acropora polystoma* (Brook, 1891) | G61679 | - | - | 1 | 1 | Intertidal |
| Acroporidae | *Acropora pulchra* (Brook, 1891) | G61082 G61682 | 7 | 5 | 7 | 19 | Intertidal |
| Acroporidae | *Acropora robusta* (Dana, 1846) | G61669 G64324 | 3 | 6 | 3 | 12 | Intertidal |
| Acroporidae | *Acropora samoensis (*Brook, 1891) | G60640 G64388 G64393 | 6 | 7 | 7 | 20 | Intertidal |
| Acroporidae | *Acropora sarmentosa* (Brook, 1892) |  | 3 | 5 | 1 | 9 | Intertidal |
| Acroporidae | *Acropora secale* (Studer, 1878) |  | 3 | - | 1 | 4 | Intertidal |
| Acroporidae | *Acropora selago* (Studer, 1878) | G60621 G60653 | 4 | 2 | 3 | 9 | Intertidal |
| Acroporidae | *Acropora solitaryensis* Veron and Wallace, 1984 |  | 1 | 1 | 2 | 4 | Intertidal |
| Acroporidae | *Acropora spicifera* (Dana, 1846) | G60624 G60625 G61083 G63139 G64392 | 4 | - | 6 | 10 | Intertidal |
| Acroporidae | *Acropora striata* (Verrill, 1866) | G60645 G61178 | 2 | - | - | 2 | Intertidal |
| Acroporidae | *Acropora subulata* (Dana, 1846) | G60629 | 3 | 5 | 2 | 10 | Intertidal |
| Acroporidae | *Acropora tenuis* (Dana, 1846) | G60630 G60655 G61666 | 4 | 7 | 5 | 16 | Intertidal |
| Acroporidae | *Acropora vaghani* Wells, 1954 | G61668 | - | 1 | - | 1 | Intertidal |
| Acroporidae | *Acropora valida* (Dana, 1846) | G60618 G60634 G60637 G60644 G60651 G61662 G61674 G61677 G64387 G64389 G64394 | 7 | 7 | 6 | 20 | Intertidal |
| Acroporidae | *Acropora verweyi* Veron and Wallace, 1984 | G60658 G60659 G61687 | 3 | 3 | 2 | 8 | Intertidal |
| Acroporidae | *Acropora yongei* Veron and Wallace, 1984 |  | - | 4 | 5 | 9 | Intertidal |
| Acroporidae | *Alveopora fenestrata* Lamarck 1816 | G64306 | - | 1 | - | 1 | Subtidal |
| Acroporidae | *Alveopora* *spongiosa* Dana, 1846 |  | 1 | 4 | - | 5 | Intertidal |
| Acroporidae | *Alveopora tizardi* Bassett-Smith, 1890 |  | - | 2 | - | 2 | Subtidal |
| Acroporidae | *Astreopora listeri* Bernard, 1896 |  | 1 | 1 | - | 2 | Intertidal |
| Acroporidae | *Astreopora myriophthalma* (Lamarck, 1816) |  | 4 | 7 | 6 | 17 | Intertidal |
| Acroporidae | *Astreopora ocellata* Bernard, 1896 | G61108 G64879 | - | 6 | - | 6 | Subtidal |
| Acroporidae | *Isopora brueggemanni* (Brook, 1893) | G60636 G61071 G61072 G61073 G61074 G64385 G65064 | 5 | 7 | 5 | 17 | Intertidal |
| Acroporidae | *Isopora palifera* (Lamarck, 1816) |  | - | 4 | 3 | 7 | Intertidal |
| Acroporidae | *Montipora aequituberculata* Bernard, 1897 | G60669 G60676 G61144 | 4 | 7 | 7 | 18 | Intertidal |
| Acroporidae | *Montipora calcarea* Bernard, 1897 | G60666 G60679 G60689 G61110 G61137 G61138 | 6 | 7 | - | 13 | Subtidal |
| Acroporidae | *Montipora caliculata* (Dana, 1846) |  | 1 | 7 | 1 | 9 | Intertidal |
| Acroporidae | *Montipora crassituberculata* Bernard, 1897 | G61129 G61130 G61131 G61132 G61133 G61588 G64308 | 3 | - | 6 | 9 | Intertidal |
| Acroporidae | *Montipora deliculata* Veron, 2000 | G61134 G61135 | 2 | - | - | 2 | Subtidal |
| Acroporidae | *Montipora digitata* (Dana, 1846) |  | - | 7 | 2 | 9 | Intertidal |
| Acroporidae | *Montipora efflorescens* Bernard, 1897 |  | - | 7 | 1 | 8 | Intertidal |
| Acroporidae | *Montipora floweri* Wells, 1954 | G64334 | 1 | - | 3 | 4 | Subtidal |
| Acroporidae | *Montipora foliosa* (Pallas, 1766) | G60668 G60671 G60680 G60688 G61141 G61142 G61143 | 6 | 2 | - | 8 | Intertidal |
| Acroporidae | *Montipora grisea* Bernard, 1897 | G60674 G60675 G61111 G61112 | 3 | 3 | - | 6 | Intertidal |
| Acroporidae | *Montipora hispida* (Dana, 1846) |  | - | 7 | - | 7 | Intertidal |
| Acroporidae | *Montipora incrassata* (Dana, 1846) | G60677 G60678 G60682 G60683 G60686 G61140 | 4 | 6 | 1 | 11 | Intertidal |
| Acroporidae | *Montipora mollis* Bernard, 1897 |  | - | 7 | - | 7 | Intertidal |
| Acroporidae | *Montipora monasteriata* (Forskäl, 1775) | G61113 | 3 | 7 | - | 10 | Intertidal |
| Acroporidae | *Montipora nodosa* (Dana, 1846) |  | - | 6 | - | 6 | Intertidal |
| Acroporidae | *Montipora spongodes* Bernard, 1897 | G64335 | - | 7 | - | 7 | Intertidal |
| Acroporidae | *Montipora tuberculosa* (Lamarck, 1816) |  | - | 7 | 4 | 11 | Intertidal |
| Acroporidae | *Montipora turgescens* Bernard, 1897 | G60690 G61125 G61127 G61128 G64323 | 4 | 7 | 6 | 17 | Intertidal |
| Acroporidae | *Montipora undata* Bernard, 1897 | G61124 | 1 | - | - | 1 | Subtidal |
| Acroporidae | *Montipora venoa* (Ehrenberg, 1834) | G61136 | 1 | 2 | 2 | 5 | Intertidal |
| Acroporidae | *Montipora verrucosa* (Lamarck, 1816) | G64290 | - | 1 | 5 | 6 | Intertidal |
| Agariciidae | *Coeloseris mayeri* Vaughan, 1918 | G61553 G61727 G61728 G64343 G64350 G64953 | 6 | 7 | 4 | 17 | Intertidal |
| Agariciidae | *Pachyseris rugosa* (Lamarck, 1801) | G64874 | 1 | 4 | - | 5 | Intertidal |
| Agariciidae | *Pachyseris speciosa* (Dana, 1846) |  | 2 | 5 | - | 7 | Intertidal |
| Agariciidae | *Pavona decussata* (Dana, 1846) | G61550 G61555 G64862 | 5 | 7 | 4 | 16 | Intertidal |
| Agariciidae | *Pavona varians* Verrill, 1864 | G64861 | 2 | 7 | 1 | 10 | Intertidal |
| Agariciidae | *Pavona venosa* (Ehrenberg, 1834) | G61551 G61552 | 4 | 7 | 4 | 15 | Intertidal |
| Astrocoeniidae | *Stylocoeniella guentheri* Bassett-Smith, 1890 |  | 1 | 2 | 1 | 4 | Subtidal |
| Coscinaraeidae | *Coscinaraea columna* (Dana, 1846) | G61725 G64309 | 1 | 4 | 2 | 7 | Intertidal |
| Coscinaraeidae | *Coscinaraea exesa* (Dana, 1846) |  | - | 3 | 3 | 6 | Intertidal |
| Dendrophylliidae | *Turbinaria bifrons* Brüggemann, 1877 | G64893 G64898 | 1 | 5 | 1 | 7 | Intertidal |
| Dendrophylliidae | *Turbinaria frondens* (Dana, 1846) | G61565 | - | - | 1 | 1 | Intertidal |
| Dendrophylliidae | *Turbinaria irregularis* Bernard 1896 | G64896 | - | 4 | - | 4 | Intertidal |
| Dendrophylliidae | *Turbinaria mesenterina* (Lamarck, 1816) | G61563 G64895 G64897 | - | 7 | 2 | 9 | Intertidal |
| Dendrophylliidae | *Turbinaria patula* (Dana 1846) | G61564 | 1 | - | - | 1 | Intertidal |
| Dendrophylliidae | *Turbinaria peltata* (Esper, 1794) | G64952 | 1 | - | 2 | 3 | Intertidal |
| Dendrophylliidae | *Turbinaria radicalis* Bernard, 1896 | G64328 | - | 6 | 2 | 8 | Intertidal |
| Dendrophylliidae | *Turbinaria reniformis* Bernard, 1896 | G64325 | 2 | 2 | 2 | 6 | Intertidal |
| Dendrophylliidae | *Turbinaria stellulata* (Lamarck, 1816) | G64894 | - | 6 | - | 6 | Intertidal |
| Diploastreidae | *Diploastrea heliopora (Lamarck, 1816)* |  | 2 | 2 | - | 4 | Intertidal |
| Euphyllidae | *Catalaphyllia jardinei* (Saville-Kent, 1893) |  | - | 1 | 5 | 6 | Subtidal |
| Euphyllidae | *Euphyllia ancora* Veron and Pichon, 1979 | G61561 G61562 | 4 | - | - | 4 | Subtidal |
| Euphyllidae | *Euphyllia glabrescens* (Chamisso and Eysenhardt, 1821) | G64890 G64892 G64899 G64945 | 4 | 3 | 2 | 9 | Intertidal |
| Euphyllidae | *Galaxea astreata* (Lamarck, 1816) | G61267 G61268 G64316 | 8 | 7 | 7 | 22 | Intertidal |
| Euphyllidae | *Galaxea fascicularis* (Linnaeus, 1767) | G64331 G64875 | 7 | 7 | 7 | 21 | Intertidal |
| Euphyllidae | *Physogyra lichtensteini* *(*Milne Edwards and Haime, 1851) | G64313 | - | 1 | - | 1 | Intertidal |
| Fungiidae | *Ctenactis crassa* (Dana, 1846) |  | - | 1 | 2 | 3 | Intertidal |
| Fungiidae | *Danafungia horrida* (Dana, 1846) |  | 1 | - | - | 1 | Intertidal |
| Fungiidae | *Fungia fungites* (Linneaus, 1758*)* | G61566 G61567 G61568 G61569 G61570 G64946 | 5 | 6 | 1 | 12 | Intertidal |
| Fungiidae | *Lithophyllon concinna* (Verrill, 1864) |  | - | 6 | - | 6 | Intertidal |
| Fungiidae | *Lithophyllon repanda* (Dana, 1846) | G61729 G64338 | 3 | 3 | 5 | 11 | Intertidal |
| Fungiidae | *Lithophyllon undulatum* Rehberg, 1892 | G61554 | 2 | 5 | - | 7 | Subtidal |
| Fungiidae | *Lobactis scutaria* (Lamarck, 1801) | G61571 | 1 | 4 | - | 5 | Intertidal |
| Fungiidae | *Podabacia crustacea* (Pallas, 1766) | G61572 G61573 G64287 G64859 G64869 | 4 | 3 | 2 | 9 | Subtidal |
| Fungiidae | *Polyphyllia talpina (Lamarck, 1801)* |  | 1 | - | 1 | 2 | Intertidal |
| Hydrozoa - Milleporina | *Millepora spp.* |  | 4 | 7 | - | 11 | Intertidal |
| Insertae sedis | *Leptastrea aequalis* Veron, 2000 | G61726 | - | 4 | - | 4 | Subtidal |
| Insertae sedis | *Leptastrea bottae* (Milne Edwards and Haime 1849) | G64300 | - | 1 | - | 1 | Subtidal |
| Insertae sedis | *Leptastrea pruinosa* Crossland, 1952 |  | 7 | - | - | 7 | Intertidal |
| Insertae sedis | *Leptastrea purpurea* (Dana, 1846) | G61723 G64326 G64345 G64348 | 7 | 7 | 4 | 18 | Subtidal |
| Insertae sedis | *Leptastrea transversa* Klunzinger, 1879 | G64315 G64333 | 6 | 6 | 2 | 14 | Intertidal |
| Insertae sedis | *Oulastrea crispata* (Lamarck, 1816) | G64943 | 2 | 2 | 2 | 6 | Subtidal |
| Insertae sedis | *Plesiastrea versipora* (Lamarck, 1816) |  | 1 | 4 | - | 5 | Intertidal |
| Lobophylliidae | *Acanthastrea bowerbanki* Milne Edwards and Haime 1851 | G61709 | - | 1 | - | 1 | Intertidal |
| Lobophylliidae | *Acanthastrea echinata* (Dana, 1846*)* | G61582 G64281 G64887 | 4 | 4 | 6 | 14 | Intertidal |
| Lobophylliidae | *Acanthastrea hemprichii* (Ehrenberg, 1834) | G61580 G61581 G64884 G64886 G64942 | 5 | 6 | 7 | 18 | Intertidal |
| Lobophylliidae | *Acanthastrea lordhowensis* Veron & Pichon, 1982 | G64327 | 2 | - | 2 | 4 | Intertidal |
| Lobophylliidae | *Echinophyllia aspera* (Ellis and Solander, 1788) | G61265 G64947 G64948 | 3 | - | 1 | 4 | Intertidal |
| Lobophylliidae | *Lobophyllia corymbosa* (Forskål, 1775) | G61583 G61694 G61695 G64291 | 6 | 4 | 7 | 17 | Intertidal |
| Lobophylliidae | *Lobophyllia diminuta* Veron, 1985 | G61578 | 2 | 6 | 5 | 13 | Subtidal |
| Lobophylliidae | *Lobophyllia flabelliformis* Veron, 2000 | G61574 G61575 | 8 | - | - | 8 | Subtidal |
| Lobophylliidae | *Lobophyllia hemprichii* (Ehrenberg, 1834) | G61577 | 8 | 7 | 7 | 22 | Intertidal |
| Lobophylliidae | *Lobophyllia serratus* (Veron 2002) | G61576 | - | 1 | - | 1 | Subtidal |
| Lobophylliidae | *Micromussa amakusensis* (*Veron 1990)* | G64885 | 1 | - | - | 1 | Intertidal |
| Lobophylliidae | *Moseleya latistellata* Quelch, 1884 | G64904 G64907 | 1 | 1 | 2 | 4 | Intertidal |
| Lobophylliidae | *Oxypora lacera* Verrill, 1864 | G61425 G64312 G64864 | 1 | - | - | 1 | Intertidal |
| Lobophylliidae | *Symphyllia agaricia* Milne Edwards and Haime, 1849 | G64889 | 5 | 7 | 4 | 16 | Subtidal |
| Lobophylliidae | *Symphyllia radians* Milne Edwards and Haime, 1849 |  | 6 | 7 | 5 | 18 | Intertidal |
| Lobophylliidae | *Symphyllia recta* (Dana, 1846) | G64888 | 8 | 7 | 8 | 23 | Intertidal |
| Lobophylliidae | *Symphyllia valenciennesi* Milne Edwards and Haime, 1849 |  | - | 7 | 5 | 12 | Intertidal |
| Merulinidae | *Astrea annuligera* (Milne Edwards and Haime, 1849) |  | 1 | 2 | 1 | 4 | Intertidal |
| Merulinidae | *Astrea curta* (Dana, 1846) | G61720 G64282 G64342 | 5 | 7 | 8 | 20 | Intertidal |
| Merulinidae | *Barabattoia amicorum* (Milne Edwards and Haime, 1850) | G61105 G61106 | 3 | - | - | 3 | Intertidal |
| Merulinidae | *Caulastrea furcata* Dana, 1846 | G61103 G64285 G64302 | 4 | - | 4 | 8 | Subtidal |
| Merulinidae | *Caulastrea tumida* Matthai, 1928 | G61097 | - | 5 | - | 5 | Intertidal |
| Merulinidae | *Coelastrea aspera* (Verrill, 1866) | G61718 G64871 G64872 G64878 G64936 | 7 | 7 | 8 | 22 | Intertidal |
| Merulinidae | *Cyphastrea chalcidium* (Forskål, 1775) | G64311 | 4 | 7 | 2 | 13 | Intertidal |
| Merulinidae | *Cyphastrea microphthalma* (Lamarck, 1816) | G61104 G64618 G64905 | 5 | 7 | 4 | 16 | Intertidal |
| Merulinidae | *Cyphastrea serailia* (Forskål, 1775) | G64310 G64321 G64332 | 2 | 7 | 3 | 12 | Intertidal |
| Merulinidae | *Dipsastraea danae* (Verrill 1872) | G61126 | - | 6 | - | 6 | Intertidal |
| Merulinidae | *Dipsastraea favus* (Forskål, 1775) | G61150 G61700 G61146 | 3 | 7 | 4 | 14 | Intertidal |
| Merulinidae | *Dipsastraea lizardensis* (Veron and Pichon, 1977) | G64317 | 1 | 1 | - | 2 | Intertidal |
| Merulinidae | *Dipsastraea maritima* (Nemenzo, 1971) | G61148 | 3 | - | 4 | 7 | Intertidal |
| Merulinidae | *Dipsastraea matthaii* (Vaughan, 1918) | G61149 G61701 G64294 G64918 G64919 | 6 | 7 | 6 | 19 | Intertidal |
| Merulinidae | *Dipsastraea maxima* (Veron, Pichon & Wijsman-Best, 1972) | G61145 | 2 | - | 1 | 3 | Intertidal |
| Merulinidae | *Dipsastraea pallida* (Dana, 1846) | G61151 G61706 G64318 G64909 G64910 G64911 | 7 | 7 | 3 | 17 | Intertidal |
| Merulinidae | *Dipsastraea rotumana* (Gardiner, 1899) |  | 6 | - | - | 6 | Intertidal |
| Merulinidae | *Dipsastraea speciosa* (Dana, 1846) | G61147 G61705 G64304 | 6 | 7 | 4 | 17 | Intertidal |
| Merulinidae | *Dipsastraea veroni* (Moll and Borel-Best, 1984) | G64314 | 1 | 5 | - | 6 | Intertidal |
| Merulinidae | *Echinopora gemmacea* Lamarck, 1816 | G61698 G64902 | 2 | 7 | 2 | 11 | Subtidal |
| Merulinidae | *Echinopora lamellosa* (Esper, 1795) | G61109 G64900 G64901 | 7 | 7 | 6 | 20 | Intertidal |
| Merulinidae | *Favites abdita* (Ellis and Solander, 1786) | G61156 G61157 G61702 G61703 G61710 G64330 G64344 G64908 G64949 G64922 | 6 | 7 | 7 | 20 | Intertidal |
| Merulinidae | *Favites chinensis* (Verrill, 1866) | G61161 G61162 G61163 G64292 | 7 | 7 | 2 | 16 | Intertidal |
| Merulinidae | *Favites complanata* (Ehrenberg, 1834) |  | 3 | 7 | 5 | 15 | Intertidal |
| Merulinidae | *Favites flexuosa* (Dana, 1846) | G61159 G61160 G64912 G64913 G64914 G64915 | 6 | 7 | 5 | 18 | Intertidal |
| Merulinidae | *Favites halicora* (Ehrenberg, 1834) | G61153 G64920 G64921 | 6 | 7 | 8 | 21 | Intertidal |
| Merulinidae | *Favites paraflexuosa* Veron, 2000 | G61152 G61704 | 1 | - | 2 | 3 | Subtidal |
| Merulinidae | *Favites pentagona* (Esper, 1794) | G64297 G64349 G64873 G64876 G64877 G64916 G64917 | 4 | 7 | 6 | 17 | Intertidal |
| Merulinidae | *Favites russelli* (Wells, 1954) |  | 1 | 7 | - | 8 | Intertidal |
| Merulinidae | *Favites stylifera* (Yabe and Sugiyama 1937) | G61158 | - | 2 | - | 2 | Subtidal |
| Merulinidae | *Favites valenciennesi* (Milne Edwards and Haime, 1848) | G64906 G64320 | 1 | 4 | 2 | 7 | Intertidal |
| Merulinidae | *Favites magnistellata* (Chevalier, 1971) |  | 4 | 7 | 4 | 15 | Intertidal |
| Merulinidae | *Favites rotundata* (Veron, Pichon & Wijsman-Best, 1972) | G64295 | 3 | 6 | 4 | 13 | Intertidal |
| Merulinidae | *Goniastrea edwardsi* Chevalier, 1971 | G64937 G64939 | 5 | 7 | 4 | 16 | Intertidal |
| Merulinidae | *Goniastrea favulus* (Dana, 1846) | G61166 G61167 G64935 | 7 | 7 | 6 | 20 | Intertidal |
| Merulinidae | *Goniastrea pectinata* (Ehrenberg, 1834) | G61164 G61165 G61716 G64283 G64284 G64617 G64932 G64933 | 5 | 7 | 4 | 16 | Intertidal |
| Merulinidae | *Goniastrea retiformis* (Lamarck, 1816) | G61075 G61076 G61078 G61079 G61080 G61715 G64319 G64868 | 7 | 7 | 7 | 21 | Intertidal |
| Merulinidae | *Goniastrea stelligera* (Dana, 1846) |  | 4 | 7 | 3 | 14 | Intertidal |
| Merulinidae | *Hydnophora exesa* (Pallas, 1766) | G61266 G61722 G64865 G64941 | 7 | 7 | 5 | 19 | Intertidal |
| Merulinidae | *Hydnophora microconos* (*Lamarck, 1816*) | G61176 | 4 | 7 | 7 | 18 | Intertidal |
| Merulinidae | *Hydnophora pilosa* Veron, 1985 |  | 3 | 5 | 1 | 9 | Intertidal |
| Merulinidae | *Hydnophora rigida* (Dana, 1846) | G61175 G64866 | 3 | 3 | 4 | 10 | Intertidal |
| Merulinidae | *Leptoria phrygia* (Ellis and Solander, 1786) | G61102 G61717 G64299 G64347 | 4 | 7 | 7 | 18 | Intertidal |
| Merulinidae | *Merulina ampliata* (Ellis and Solander, 1786) | G61177 | 4 | 6 | 3 | 13 | Intertidal |
| Merulinidae | *Merulina scabricula* Dana, 1846 |  | 6 | 4 | 4 | 14 | Subtidal |
| Merulinidae | *Mycedium elephantotus* (Pallas, 1766) | G61426 G61711 G64940 | 6 | 1 | 2 | 9 | Intertidal |
| Merulinidae | *Oulophyllia bennettae* (Veron & Pichon, 1977) |  | 3 | 1 | 1 | 5 | Subtidal |
| Merulinidae | *Oulophyllia crispa* (Lamarck, 1816) | G61707 G61708 G64857 G64858 G64903 G64944 | 2 | 4 | 3 | 9 | Intertidal |
| Merulinidae | *Paragoniastrea australensis* (Milne Edwards, 1857) | G61077 G61719 G64329 G64870 G64934 | 4 | 7 | 5 | 16 | Intertidal |
| Merulinidae | *Pectinia lactuca* (Pallas, 1766) | G61263 | 3 | 2 | 1 | 6 | Subtidal |
| Merulinidae | *Pectinia paeonia* (Dana, 1846) | G61264 G61712 G64867 G64951 G64860 | 3 | 5 | 1 | 9 | Intertidal |
| Merulinidae | *Platygyra acuta* Veron, 2000 | G64950 | 6 | 5 | 5 | 16 | Intertidal |
| Merulinidae | *Platygyra carnosus* Wijsman-Best, 1976 | G61170 | - | - | 5 | 5 | Subtidal |
| Merulinidae | *Platygyra daedalea* (Ellis and Solander, 1786) | G61168 G61169 G64926 G64927 G64928 G64929 G64930 | 6 | 7 | 6 | 19 | Intertidal |
| Merulinidae | *Platygyra lamellina* (Ehrenberg, 1834) | G61171 | 5 | 7 | 3 | 15 | Intertidal |
| Merulinidae | *Platygyra pini* Chevalier, 1975 | G61714 | 7 | 7 | 3 | 17 | Intertidal |
| Merulinidae | *Platygyra ryukyuensis* Yabe and Sugiyama, 1936 | G64931 | 5 | 7 | 6 | 18 | Subtidal |
| Merulinidae | *Platygyra sinensis* (Milne Edwards and Haime, 1849) | G61172 G61173 G61174 G61713 G64924 G64925 G64923 | 7 | 7 | 7 | 21 | Intertidal |
| Octocorallia - Alcyonacea | *Tubipora musica* (Linnaeus 1758) | G61107 | 4 | 7 | 6 | 17 | Intertidal |
| Octocorallia - Helioporacea | *Heliopora coercula* (Pallas, 1766) |  | 6 | 7 | 4 | 17 | Intertidal |
| Pocilloporidae | *Pocillopora damicornis* (Linnaeus, 1758) | G64341 G64863 | 8 | 7 | 8 | 23 | Intertidal |
| Pocilloporidae | *Pocillopora* *meandrina* Dana, 1846 |  | 2 | 2 | - | 4 | Intertidal |
| Pocilloporidae | *Pocillopora verrucosa* (Ellis and Solander, 1786) | G64881 G64891 | 4 | 3 | 1 | 8 | Intertidal |
| Pocilloporidae | *Seriatopora aculeata* Quelch, 1886 | G64619 | 4 | 1 | 4 | 9 | Intertidal |
| Pocilloporidae | *Seriatopora caliendrum* Ehrenberg, 1834 | G61556 G61557 | 5 | 1 | 6 | 12 | Intertidal |
| Pocilloporidae | *Seriatopora hystrix* Dana, 1846 | G61558 G64296 G64298 | 6 | 7 | 7 | 20 | Intertidal |
| Pocilloporidae | *Stylophora pistillata* Esper, 1797 | G61559 G61560 G64305 G64883 | 8 | 6 | 8 | 22 | Intertidal |
| Poritidae | *Goniopora columna* Dana, 1846 |  | 3 | 7 | - | 10 | Subtidal |
| Poritidae | *Goniopora djiboutiensis* Vaughan, 1907 | G60673 G61724 | 2 | - | 1 | 3 | Intertidal |
| Poritidae | *Goniopora fruticosa* Saville-Kent 1893 | G64340 | - | 1 | - | 1 | Intertidal |
| Poritidae | *Goniopora lobata* Milne Edwards, 1860 |  | - | 7 | 5 | 12 | Intertidal |
| Poritidae | *Goniopora norfolkensis* Veron and Pichon 1982 | G64346 | - | 1 | - | 1 | Intertidal |
| Poritidae | *Goniopora pendulus* Veron, 1985 |  | - | 4 | - | 4 | Subtidal |
| Poritidae | *Goniopora somaliensis* Vaughan, 1907 | G61118 G64938 | 3 | 7 | 2 | 12 | Intertidal |
| Poritidae | *Goniopora tenuidens* (Quelch, 1886) | G60667 G60670 G60684 G60685 G60687 G61117 | 3 | 1 | 6 | 10 | Intertidal |
| Poritidae | *Porites annae* Crossland, 1952 |  | 5 | 1 | - | 6 | Intertidal |
| Poritidae | *Porites aranetai* Nemenzo, 1955 | G61114 G61115 | 3 | 2 | - | 5 | Intertidal |
| Poritidae | *Porites cylindrica* Dana, 1846 | G61119 G61120 G61121 G61696 | 6 | 7 | 5 | 18 | Intertidal |
| Poritidae | *Porites lichen* Dana, 1846 |  | 2 | - | 2 | 4 | Intertidal |
| Poritidae | *Porites lobata* Dana, 1846 | G61122 G61123 | 7 | 7 | 4 | 18 | Intertidal |
| Poritidae | *Porites lutea* Milne Edwards & Haime, 1851 |  | 3 | 7 | 7 | 17 | Intertidal |
| Poritidae | *Porites nigrescens* Dana, 1846 | G61098 G61099 G61100 | 1 | 5 | - | 6 | Intertidal |
| Poritidae | *Porites rus* (Forskål, 1775) | G61101 | 4 | 7 | - | 11 | Intertidal |
| Poritidae | *Porites solida* (Forskål, 1775) |  | - | 3 | - | 3 | Subtidal |
| Poritidae | *Stylaraea punctata* (Linneaus, 1758) | G60681 | 1 | - | - | 1 | Subtidal |
| Psammocoridae | *Psammocora contigua* (Esper, 1797) | G61697 | 4 | 7 | 5 | 16 | Intertidal |
| Psammocoridae | *Psammocora digitata* Milne Edwards and Haime, 1851 | G61548 G61699 G64289 | - | 3 | - | 3 | Intertidal |
| Psammocoridae | *Psammocora haimiana* Milne Edwards and Haime, 1851 |  | - | 1 | 1 | 2 | Subtidal |
| Psammocoridae | *Psammocora profundacella* Gardiner, 1898 | G64301 G64303 G61549 G64288 | 3 | 7 | 3 | 11 | Intertidal |
| Siderastreidae | *Pseudosiderastrea tayami* Yabe and Sugiyama, 1935 | G64286 | - | 4 | 4 | 8 | Intertidal |
|  | **Total Species** |  | **176** | **189** | **162** | **225** |  |

**Table E.** Summary of significance results from one-way analysis of variance comparing the mean SST and Kd(490) time-series data between locations.

| **SST** | **North Maret Island** | **Scott Reef** | **Barrow Island** | **Lizard Island** | **Dent Island** |
| --- | --- | --- | --- | --- | --- |
| **North Maret Island** |  | **ns** | *** | *** | *** |
| **Scott Reef** | *** |  | *** | *** | *** |
| **Barrow Island** | *** | *** |  | * | *** |
| **Lizard Island** | *** | *** | *** |  | *** |
| **Dent Island** | **ns** | *** | *** | *** |  |
| **Kd490** | **North Maret Island** | **Scott Reef** | **Barrow Island** | **Lizard Island** | **Dent Island** |

ns = non –significant; * = <0.05; **p<0.005; *** p<0.0005


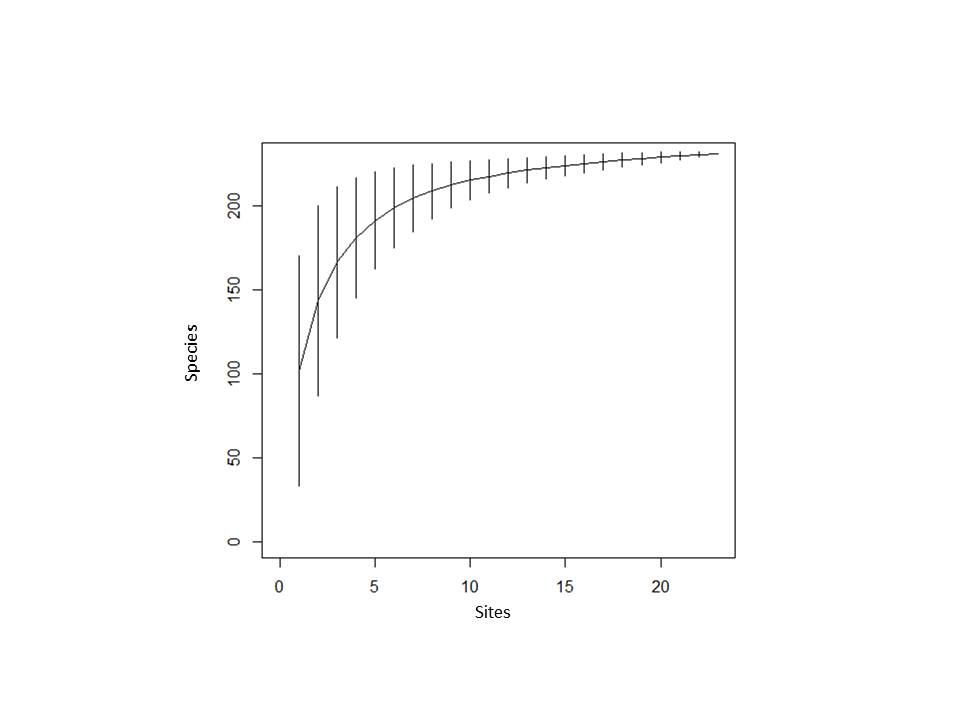


**Figure A.** Permutated species accumulation curves indicate the local species diversity was adequately surveyed after approximately 20 sites were surveyed in the Bonaparte Archipelago.


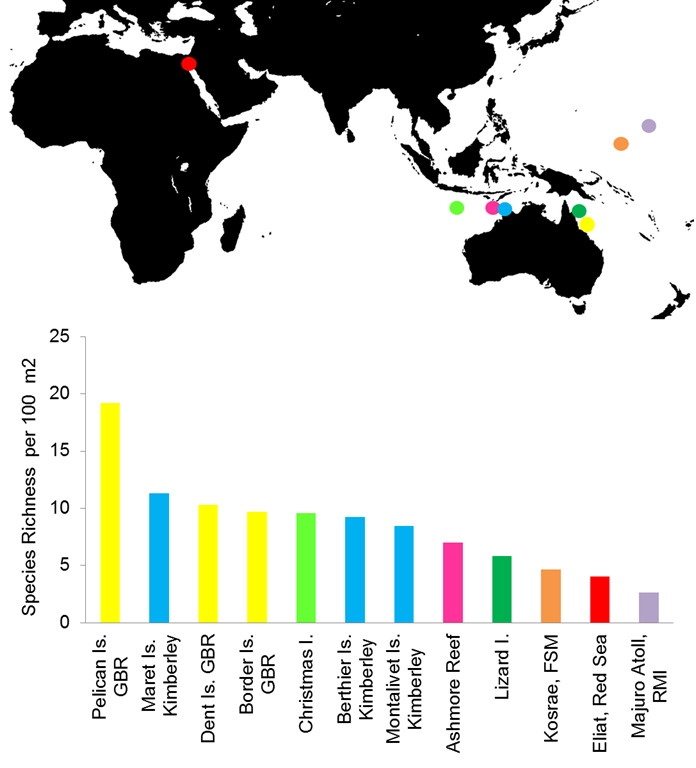


**Figure B.** **Spatial comparison of coral species diversity.** This figure illustrates that the three Bonaparte Island groups (Maret I., Berthier I., and Montalivet I., *in blue*) have a similar level of diversity to other more typical and less physically extreme reef locations such as Dent Island and Border Island on the Great Barrier Reef and Christmas Island, an offshore oceanic location in the NE Indian Ocean. The level of diversity per 100m^2^ is higher than Ashmore Reef (Offshore Kimberley); Lizard Island (Northern GBR); Kosrae and Majuro (Central Pacific) and the Red Sea. Data summarized from [37], [72-77].

**
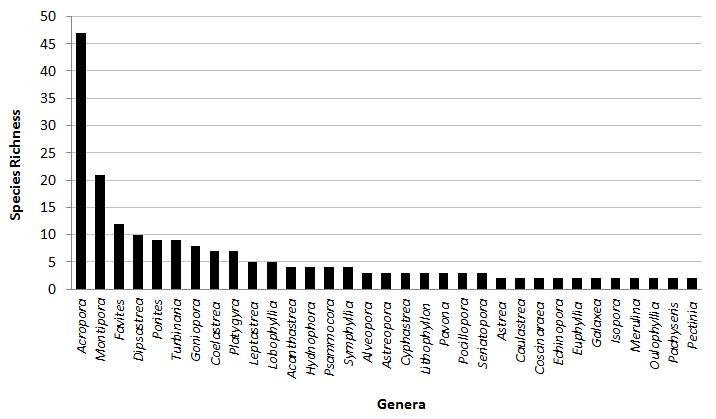
**

**Figure C.** **Species level diversity within genera at the 23 intertidal survey sites**. Note: 27 genera were represented by a single species (Table D in S1 File).


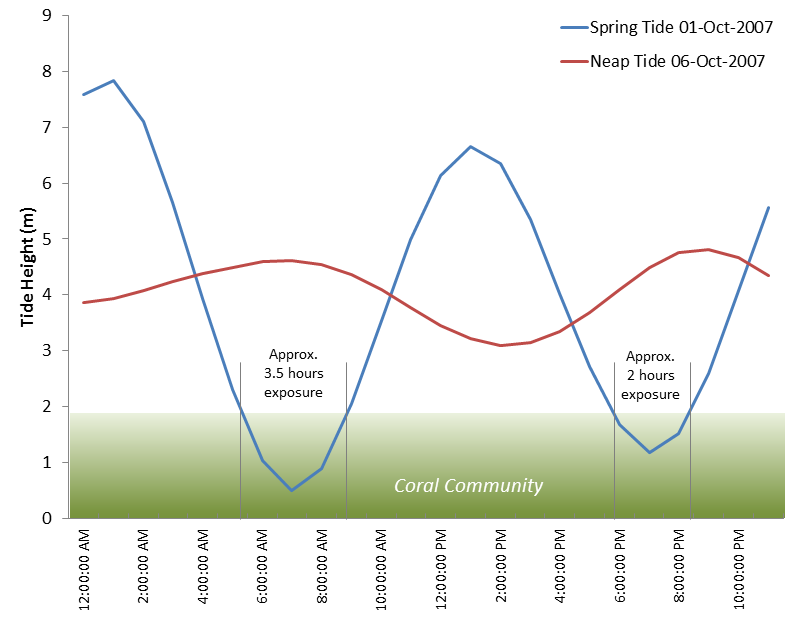


**Figure D. Daily tidal cycle at North Maret Island on selected spring and neap tides over our survey period in October 2007.** During spring low tides (i.e. tides ≤ 2m), corals growing on the intertidal reef platform at North Maret Island are exposed to the air for up to 3.5 hours at a time whereas at neap tide, corals remain submerged by at least 1m of water. See Figure 5 for a time-series analysis showing the proportion of tides occurring at 1m intervals from 0-1m up to 7-8m.


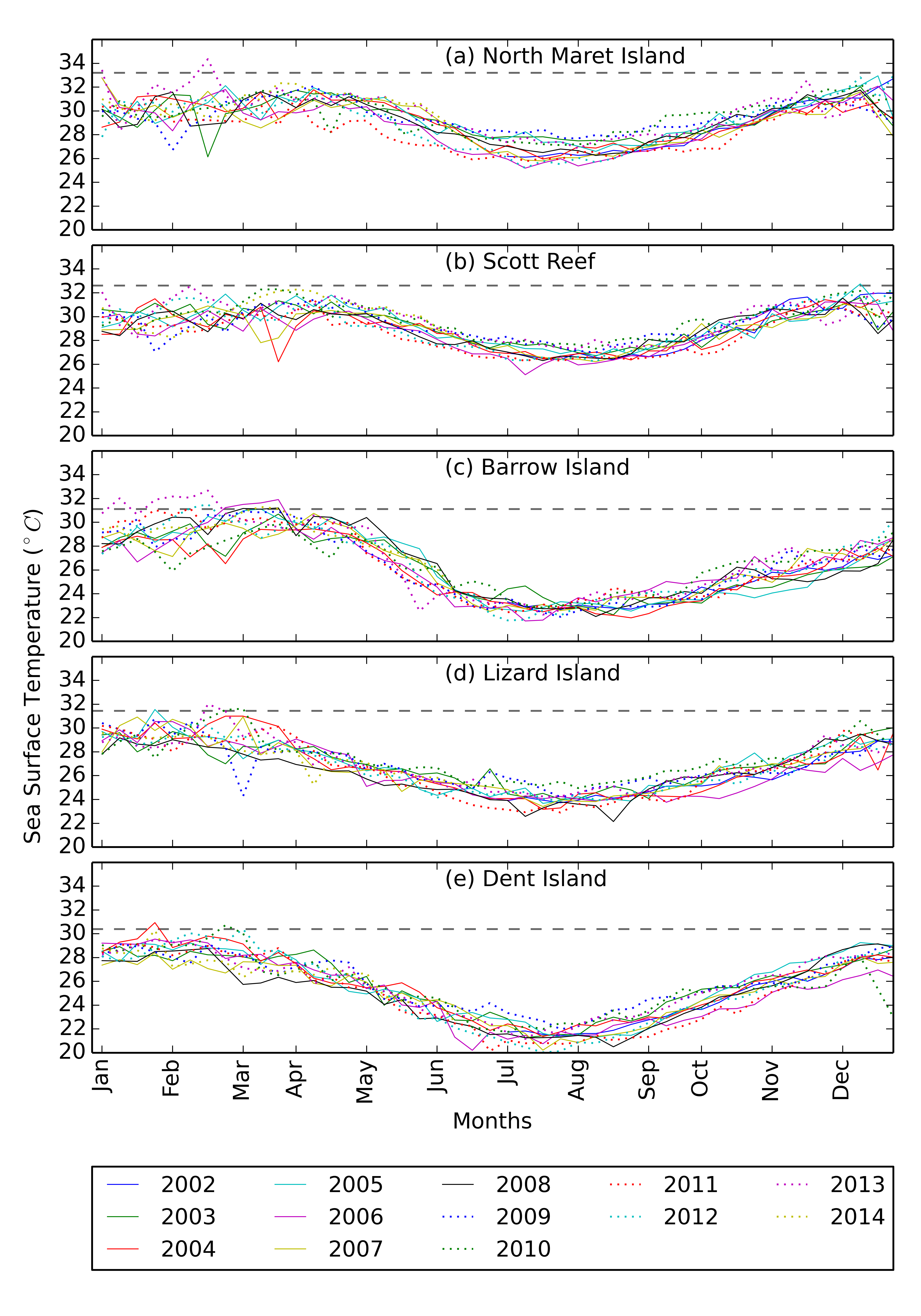


**Figure E. Time series (2002-2014) showing SST data based on** 8**-day averages for 5 locations.** This figure shows at North Maret Island SST surpassed the +1°C bleaching threshold in Feb-March 2013.


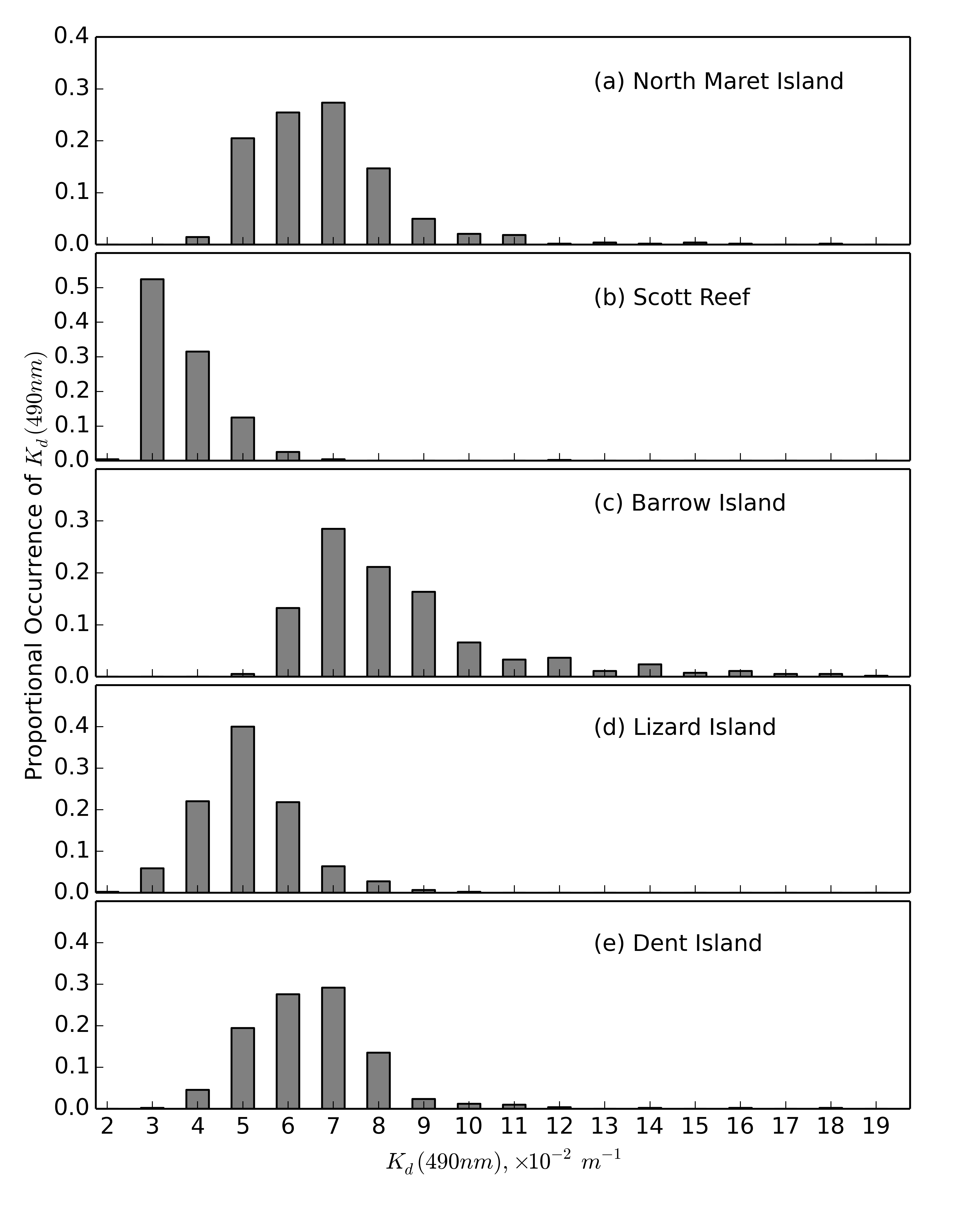


**Figure F**. **Spatial comparison of K_d_490 – 2002-2014.** K_d_(490) represents the diffuse attenuation coefficient of down-welling irradiance at 490 nm and is used as a measure of the turbidity of the water.

**
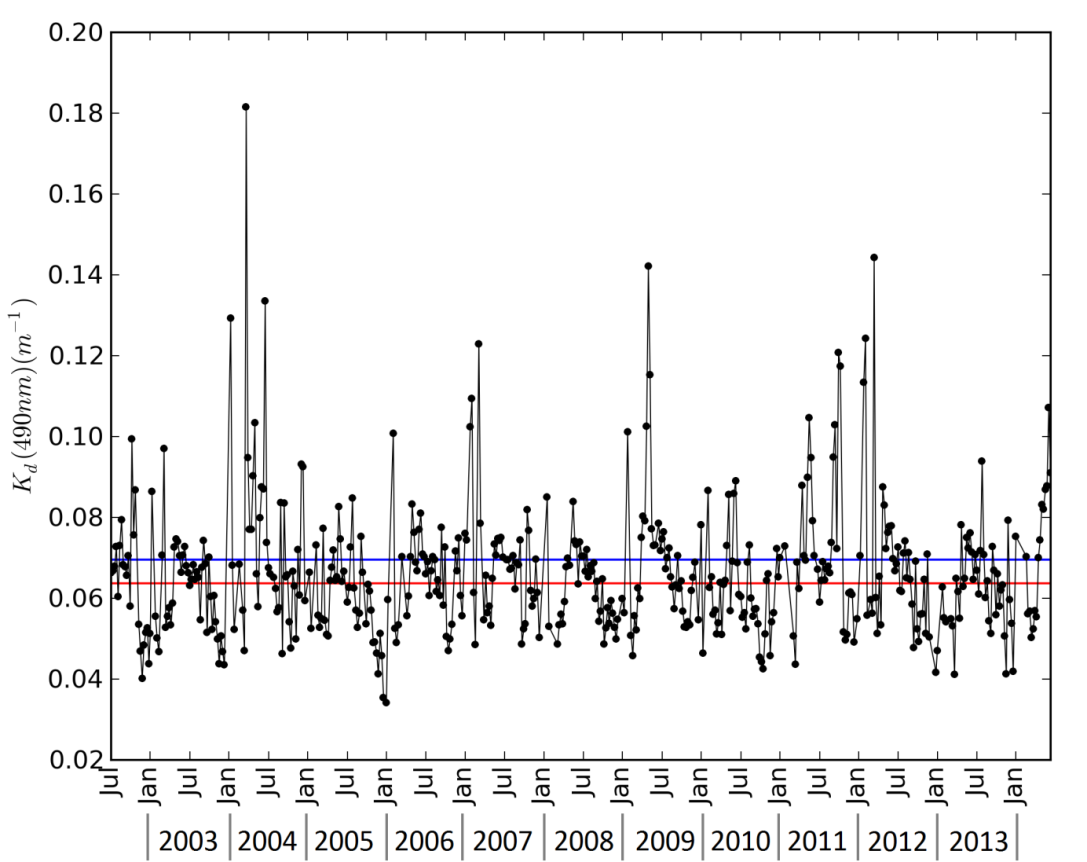
**

**Figure G.** **K_d_(490) time series for North Maret Island from 2002-2014**. The blue line represents the average winter turbidity level and the red represents the average summer turbidity.
